# Isolation, characterization, and functional study of extracellular vesicles derived from *Leishmania tarentolae*

**DOI:** 10.3389/fcimb.2022.921410

**Published:** 2022-08-03

**Authors:** Mehrdad Shokouhy, Hamzeh Sarvnaz, Yasaman Taslimi, Mahya Sadat Lajevardi, Sima Habibzadeh, Amir Mizbani, Faezeh Shekari, Mandana Behbahani, Ana Claudia Torrecilhas, Sima Rafati

**Affiliations:** ^1^ Department of Immunotherapy and Leishmania Vaccine Research, Pasteur Institute of Iran, Tehran, Iran; ^2^ Department of Biotechnology, Faculty of Biological Science and Technology, University of Isfahan, Isfahan, Iran; ^3^ Department of Health Science and Technology, Eidgenössische Technische Hochschule (ETH) Zurich, Zurich, Switzerland; ^4^ Department of Stem Cells and Developmental Biology Cell Science, Research Center, Royan Institute for Stem Cell Biology and Technology, Academic center tor Education, Culture and Research (ACECR), Tehran, Iran; ^5^ Laboratório de Imunologia Celular e Bioquímica de Fungos e Protozoários, Departamento de Ciências Farmacêuticas, Universidade Federal de São Paulo (UNIFESP), Diadema, Brazil

**Keywords:** *Leishmania tarentolae*, *Leishmania* major, extracellular vesicles, human macrophage cell line, infection

## Abstract

*Leishmania* (*L.*) species are protozoan parasites with a complex life cycle consisting of a number of developmental forms that alternate between the sand fly vector and their host. The non-pathogenic species *L. tarentolae* is not able to induce an active infection in a human host. It has been observed that, in pathogenic species, extracellular vesicles (EVs) could exacerbate the infection. However, so far, there is no report on the identification, isolation, and characterization of *L. tarentolae* EVs. In this study, we have isolated and characterized EVs from *L. tarentolae* ^GFP+^ (tEVs) along with *L. major* ^GFP+^ as a reference and positive control. The EVs secreted by these two species demonstrated similar particle size distribution (approximately 200 nm) in scanning electron microscopy and nanoparticle tracking analysis. Moreover, the said EVs showed similar protein content, and GFP and GP63 proteins were detected in both using dot blot analysis. Furthermore, we could detect *Leishmania*-derived GP63 protein in THP-1 cells treated with tEVs. Interestingly, we observed a significant increase in the production of IFN-γ, TNF-α, and IL-1β, while there were no significant differences in IL-6 levels in THP-1 cells treated with tEVs following an infection with *L. major* compared with another group of macrophages that were treated with *L. major* EVs prior to the infection. Another exciting observation of this study was a significant decrease in parasite load in tEV-treated *Leishmania*-infected macrophages. In addition, in comparison with another group of *Leishmania*-infected macrophages which was not exposed to any EVs, tEV managed to increase IFN-γ and decrease IL-6 and the parasite burden. In conclusion, we report for the first time that *L. tarentolae* can release EVs and provide evidence that tEVs are able to control the infection in human macrophages, making them a great potential platform for drug delivery, at least for parasitic infections.

## Introduction

Leishmaniasis can be categorized into four types: visceral leishmaniasis (VL), cutaneous leishmaniasis (CL), mucocutaneous leishmaniasis (MCL), and diffuse cutaneous leishmaniasis (DCL). Leishmaniasis has an approximate annual incidence of one million cases in 98 tropical and subtropical countries. These *Leishmania*-endemic countries accommodate over one billion people, where VL solely causes about 30,000 deaths every year. CL, on the other hand, puts an estimated number of 400 million people at risk in endemic regions (https://www.who.int/en/news-room/factsheets/detail/leishmaniasis; Alvar et al., 2012; Castelli et al., 2019). Despite the recent advances in anti-leishmanial chemotherapeutics, there are major concerns about chemotherapy approaches (Gannavaram et al., 2019).

The protozoan parasites of the genus *Leishmania* belong to the Trypanosomatidae family (one of the oldest groups of eukaryotes), which comprises unicellular organisms recognized by their single flagellum and a unique mitochondrial DNA-containing organelle, the kinetoplast (Niimi, 2016). Due to its public health significance, the Trypanosomatidae group is one of the best-studied eukaryotic groups after yeast (Basile and Peticca, 2009). *Leishmania* are obligate intracellular parasites with a dual life cycle that involves several developmental forms, occurring either in the midgut of the sand fly vector or in mammalian host macrophages (Atayde et al., 2015; Atayde et al., 2019). The parasite replicates as extracellular and motile flagellated cells known as promastigotes, which reside in the insect’s alimentary tract (Besteiro et al., 2007). In contrast with the pathogenic *Leishmania* species as mentioned above, *L. tarentolae*, a trypanosomatid protozoan parasite of the white-spotted wall gecko (*Tarentola annularis*), is a non-pathogenic species and absolutely safe for mammals since its natural hosts are lizards.

*L. tarentolae* does not cause pathogenicity in humans and even in severe combined immunodeficient mice (Breton et al., 2005; Niimi, 2016). Recently, many studies on animals, such as mice, hamsters, and dogs have shown that the use of *L. tarentolae* can be a promising approach for vaccine development (as a live vector) against CL and VL (Saljoughian et al., 2013; Zahedifard et al., 2014). *L. tarentolae* can infect macrophages and dendritic cells, but its survival and propagation in mammalian cells are limited (Breton et al., 2005). Furthermore, *L. tarentolae* can act as an efficient delivery system due to its unique features that have the ability to produce mammalian-like complex N-glycosylation patterns, the ease of genetic manipulation, the straightforward adaptation to large-scale production, the minimal nutrition requirements, and, most importantly, the ability to target dendritic cells and secondary lymphoid organs and to create Th1 responses and IFN-γ production (Basile and Peticca, 2009; Mizbani et al., 2009; Bolhassani et al., 2011; Saljoughian et al., 2013; Zahedifard et al., 2014; Katebi et al., 2015; Niimi, 2016; Abdossamadi et al., 2017).

The International Society of Extracellular Vesicles endorses extracellular vesicles (EVs) as the generic term for particles naturally released from all cell types (Cocucci and Meldolesi, 2015; Théry et al., 2018). EVs are surrounded by a lipid bilayer and cannot replicate due to the lack of a functional nucleus (Théry et al., 2018). The term EV refers to all sub-populations of particles, such as endosome-origin “exosomes” and plasma membrane-derived “ectosomes” (microvesicles) and apoptotic bodies (Cocucci and Meldolesi, 2015; Théry et al., 2018; Torrecilhas et al., 2020). Many different cell types have been observed to use EVs as vehicles for delivery of modulatory proteins, lipids, and nucleic acids to short- and long-distance cells (Szempruch et al., 2016; Maas et al., 2017). Initially, it was reported that *Leishmania* and *Trypanosoma* parasites require an unconventional protein secretion system to release proteins without a signal peptide, and EV biogenesis pathways were considered as the underlying mechanism to explain this incidence (Silverman et al., 2008; Cuervo et al., 2009; Théry et al., 2009; Torrecilhas et al., 2009; Corrales et al., 2010; Geiger et al., 2010; Silverman et al., 2010; Hassani et al., 2011). To date, the great majority of *Leishmania* species have been studied, and it is shown that only 5–9% of proteins present in *Leishmania* EVs have a signal peptide (Torrecilhas et al., 2020). It is widely appreciated that *Leishmania* secrete bioactive compounds that are involved in pathogenesis (Dong and Olivier, 2019). Generally, the EV cargo of pathogenic *Leishmania* species mediates immunosuppression and functionally primes the host cells for *Leishmania* infection. Moreover, it has been reported that various virulence factors are delivered to the host cells by these EVs. This can be concluded from studies in mice and macrophages that have reported that *Leishmania* EVs modulate immune-regulating and signaling pathways (Silverman et al., 2010; Silverman and Reiner, 2012; Atayde et al., 2015; Atayde et al., 2016). However, all these studies have focused on pathogenic species of *Leishmania* so far, and there is no evidence for *L. tarentolae* (as a non-pathogenic *Leishmania* sp.) to be pathogenic or have any exacerbatory effect on previously infected dogs (Mendoza-Roldan et al., 2021).

To the best of our knowledge, there is no report about *L. tarentolae* having exosomes or microparticles in general. Therefore, one of the main goals of this study is to examine whether this species secretes EVs. Raymond, Frédéric, *et al.* have briefly commented on the exosome biogenesis pathway in *L. tarentolae*, as they provided the first whole-genome sequence of *L. tarentolae*, and could not find some of the genes previously reported to be involved in this pathway, such as some of the adaptin subunits as well as Arp2/3 complex (Raymond et al., 2012). However, the pathway for the biogenesis of exosomes is very complex, and a sequence-based homology search within the genome sequence might not give the definitive answer as to whether *L. tarentolae* is able to generate and secrete EVs. One possible scenario, for example, is that the pathway is slightly different in *L. tarentolae*, or other genes with different sequences compensate for the seemingly missing genes. This is corroborated by the fact that less than half of the *Leishmania* genome is annotated, so the function of the majority of its genes is not known. On the other hand, discovering *L. tarentolae* EV (tEV) can be a huge step forward and probably will open up a new field in the therapeutic applications of *L. tarentolae*, such as vaccine development, delivery of drugs, and therapeutic proteins for further immunostimulation since *L. tarentolae* is completely safe and not pathogenic to humans (Dong et al., 2021).

## Materials and methods

### Materials and chemicals

All solutions were prepared by apyrogenic deionized water (MilliQ System, Millipore, France). All materials used for real-time PCR were purchased from Qiagen (Germany), except the DNA extraction kit that was GF-1 Nucleic Acid Extraction Kit and was purchased from Vivantis Technologies (Malaysia). The GP63 antibody was provided by the Pasteur Institute of Iran, the anti-GFP-HRP goat polyclonal antibody was obtained from Acris antibodies GmbH (Germany), and the beta actin monoclonal antibody was purchased from ProteinTech Group Inc. (Chicago, IL, USA). The nitrocellulose membrane was supplied from Amersham (UK), and the ECL Western blotting substrate was from Pierce™ (Pierce Biotechnology, Thermo Fisher, Rockford, IL, USA). The bovine serum albumin (BSA), phorbol 12-myristate 13-acetate (PMA), Resazurin powder, M199 and RPMI-1640 cell culture media, HEPES, L-glutamine, adenosine, gentamicin, and hemin were sourced from Sigma (Germany). The fetal bovine serum (FBS) and EV-depleted FBS were obtained from Gibco (Life Technologies, Germany). The bicinchoninic acid (BCA) and ELISA kits were purchased from Pierce™ (BCA Protein Assay Kit, USA) and DuoSet ELISA development system (R&D Systems, USA), respectively.

### Parasites

*L. tarentolae* ^GFP+^ (ATCC 30267) and *L. major* ^GFP+^ (MRHO/IR/75/ER) (Bolhassani et al., 2011) promastigote forms were grown at 26°C for 4 days in M199 medium supplemented with 5 and 10% heat-inactivated fetal bovine serum, respectively, and 40 mM HEPES, 2 mM L-glutamine, 0.1 mM adenosine, 0.5 µg/ml hemin, and 50 µg/ml gentamicin to reach a late-log phase (**Supplementary Figure S1**). For all THP-1 macrophage infections, stationary-phase *L. major* ^GFP+^ promastigotes were utilized.

### Viability assessment by Resazurin

The parasite’s viability was assessed using Resazurin solution according to the manufacturer’s instruction. Briefly, 6.2mg of Resazurin powder was dissolved in 50 ml PBS and passed through a 0.22-µm sterile syringe filter. A total volume of 20 µl of Resazurin solution was added to 10^7^ parasites (**Supplementary Figure S2**) or 5 × 10^5^ THP-1 cells diluted in 180 µl of M199 or RPMI1640, respectively, in a 96-well maxi sorb plate (SPL). Then, the plate was incubated for 4 hours at 26° or 37°C. The percentage of the parasite’s viability was evaluated at 540 nm (with wavelength correction set at 630 nm) on an ELISA plate reader (Sunrise, TECAN, Switzerland), as shown in **Supplementary Table S1**.

### Isolation of EVs by size exclusion chromatography

*Leishmania* late-log phase promastigotes were washed three times with sterile PBS and resuspended in M199 medium without FBS. Approximately 10^8^ promastigotes were placed in each microtube and incubated for 4 h at 37° or 26°C for *L. major* ^GFP+^ or *L. tarentolae* ^GFP+^, respectively, to release EVs in the culture medium. Parasite viability was measured by Resazurin before and after incubation for 4 h. At the end of 4 h of incubation, the samples were centrifuged at 1,800 g, and the supernatants were filtered through 0.45-µm sterile syringe filters and subjected to serial centrifugation at 4°C as follows: 500 g for 10 min, 1,500 *g* for 10 min, and 10,000 *g* for 10 min. Then, the supernatant of the last step of serial centrifugation was concentrated using Amicon Ultra-15-3K and passed through a 0.5-ml qEV size-exclusion chromatography column (Izon Science, Christchurch, New Zealand) according to the manufacturer’s protocol. The final products (purified EVs) derived from *L. major* ^GFP+^ and *L. tarentolae* ^GFP+^ are herein referred to as mEV and tEV, respectively (**Supplementary Figure S1**).

### THP-1 macrophages

The human pro-monocytic cell line THP-1 (ATCC^®^ TIB-202™) was first stimulated and differentiated by 5 µg/ml of PMA in RPMI medium with 10% EV-depleted FBS (EFBS) to adhere to the cell culture plate wells overnight. All incubations were performed at 37°C in 5% CO_2_ for 24, 48, and 72 h. For the assessment of the impact of EV-depleted FBS on THP-1 propagation, THP-1 macrophages were cultured with PMA after overnight incubation at 37°C in a CO_2_ incubator on a flat-bottomed 96-well plate. The cells were then washed twice with warm sterile PBS, and 180 μl of RPMI culture medium containing 10% EFBS was added to each well and incubated overnight. After incubation, 20 μl of Resazurin solution was added to each of the test wells and incubated for 4 h. Afterwards, the amount of light absorption at 540 nm (reference wavelength = 630 nm) in the wells for the period of interest was read using an ELISA reader (Sunrise, TECAN, Switzerland).

### Bicinchoninic acid and dot blot

The protein concentrations of mEV, tEV, and cell lysate of parasites and macrophages (as a control for some experiments) were estimated using BCA protein assay kit (Pierce™, USA) according to the manufacturer’s instruction. Dot blots were performed with EVs (50 µl or 1 µg) or *L. major* ^GFP+^ and *L. tarentolae* ^GFP+^ extract (35 µg each, 1,100 and 1,445 µg/ml, respectively) applied to the nitrocellulose membrane. After drying out, the membranes were blocked overnight with 2.5% BSA in TBST at 4°C and washed three times with 10% TBS containing 0.1% Tween 20. Then, the membranes were probed by anti-GP63 monoclonal antibody (1:5) or anti-GFP antibody (1:5,000) or anti-β-actin antibody (1:10,000) for 2 h at room temperature. The membranes were washed three times and then incubated with goat anti mouse IgG conjugated with peroxidase (1:5,000) (the secondary antibody was used only for anti-GP63 and anti-β-actin since anti-GFP was HRP-conjugated). After washing, the reaction was visualized using ECL western blotting substrate. The membranes were exposed to ECL western blotting substrate for 5 min.

### Nanoparticle tracking analysis

The EVs isolated from parasites were diluted 3–5 times and submitted to nanoparticle tracking analysis (NTA) by using a NanoSight NS300 equipment (Malvern Panalytical, Ltd.) coupled to a sCMOS camera at 532-nm wavelength. The camera level was set to auto, the detection threshold was set to 10, and the focus was set in manual set. A volume of 500 µl/sample was manually injected, and readings were taken in triplicate for 30 s at 25 frames per second. The laser chamber was cleaned with distilled water between each sample, and the data was analyzed using NTA software (NTA version 3.2 Dev Build 3.2.16). The amount of protein contamination present in each EV sample was determined by calculating the ratio of particle count and protein concentration (Webber and Clayton, 2013) in order to assess the level of contaminating non-EV protein retrieved from each species.

### Scanning electron microscopy and fluorescent microscopy

The parasites were incubated for 4 h in serum-free media, fixed in 2.5% glutaraldehyde solution at 4°C, added to the slides coated with polylysine (Sigma, Germany), and dehydrated in ethanol. The dried samples were coated with Pd–Au and imaged using an electron microscope (TESCAN, Czech Republic). As the parasites were able to express GFP, their morphology was precisely evaluated before and after the 4 h incubation using fluorescent microscopy at ×100 magnification (Nikon ECLIPSE E200, Japan).

### THP-1 cell line infection

In total, 5 × 10^5^ THP-1 cells per well were harvested and seeded in RPMI1640 containing 10% EFBS in 48-well flat-bottomed culture plates (SPL, Korea) with 5 μg/ml PMA overnight. The cells were washed with warm PBS to remove any unattached cells or PMA. Afterwards, tEV or mEV (1 μg diluted in 50 μl PBS, ~3 × 10^7^ particles per well) was added to each well and incubated for 24 h at 37°C in 5% CO_2_. After the cells were washed, 5 × 10^5^ stationary phase promastigotes were added to the cells and incubated for 6 h. Three different groups of seeded THP-1 cells were used as controls: one group was only infected with *L. major* ^GFP+^ and did not receive any EV as positive control, another group was not infected with *L. major* ^GFP+^ and just treated with tEV or mEV, and the last group was not treated or infected as the negative control. Following another washing step, the cells were collected after 24 h and 72 h.

### Cytokine measurements

For the ELISA-based cytokine assay, THP-1 cell supernatants were collected at different time points after infecting the cells with stationary phase *L. major* ^GFP+^ for 6 h (except for the negative and positive controls). Then, after three washes of all wells (including positive and negative controls), the first supernatant collection started after 24 h for TNF-α and 48 h for IL-6, and the last collection was for IFN-γ and IL-1β at 72 h. All collected supernatants were kept at -70°C until use. Afterwards, the cytokines were measured using cytokine assay kits (DuoSet, R&D Systems, USA) according to the manufacturer’s specifications. The detection range of the aforementioned kits was 9–600 pg/ml for IFN-γ and IL-6, 3–250 pg/ml for IL-1β, and 15.6–1,000 pg/ml for TNF-α.

### Quantification of parasite burden in the infected THP-1 cells by real-time PCR

The genomic DNA (gDNA) of THP-1 cells (the test group and the three different control groups mentioned above) were extracted using GF-1 Nucleic Acid Extraction Kit according to the manufacturer’s instructions. The concentration and purity of the extracted DNA was assessed using a Nanodrop (ND-1000) spectrophotometer. Serial dilutions of *L. major* gDNA in the range of 10 to 10 million parasites were used to generate the standard curve. Each PCR reaction consisted of 40 ng DNA, 5 pmol from each primer RV1 and RV2 (forward: 5′- CTTTTCTGGTCCCGCGGGTAGG-3′; reverse: 5′-CCACCTGGCCTATTTTACACCA-3′; Sigma-Aldrich), 5 μl of 2X SYBR green PCR master mix, and 1 μl of QN Rox dye (Qiagen, Germany) in a total volume of 10 μl. The PCR program consisted of an initial denaturation step at 95°C for 20 s, followed by 40 cycles of 95°C for 3 s and 60°C for 30 s. The reactions were performed in triplicates, and a no-template control was included in the plate as well. PCR amplifications and melt curve analysis were performed on StepOne Plus Real-Time PCR instrument (software v. 2.3; Applied Biosystems, USA) (Seif et al., 2016; Khadir et al., 2018).

### Preparation of parasite and macrophage lysate

A total of 10^8^ stationary phase parasites per milliliter were subjected to repeated freezing in liquid nitrogen and thawing in a 37°C water bath until the parasites were lysed completely (20–22 cycles). These repeated cycles were done on 5 × 10^5^ THP-1 macrophages for five cycles. The protein content of the lysates was quantitated using a Pierce BCA Protein kit.

### Statistical analysis

Statistical analysis was accomplished using Graph-Pad Prism (version 8.4.3). All values are presented as mean ± SD, and the statistical comparison of the two or more data sets was performed using one-way ANOVA with *post-hoc* Tukey’s test. A *p-value* less than 0.05 was considered statistically significant. All indicated data are representative of at least two individual experiments.

## Results

### Intact morphology of parasites after 4 h in serum-free media

**Figure 1:**
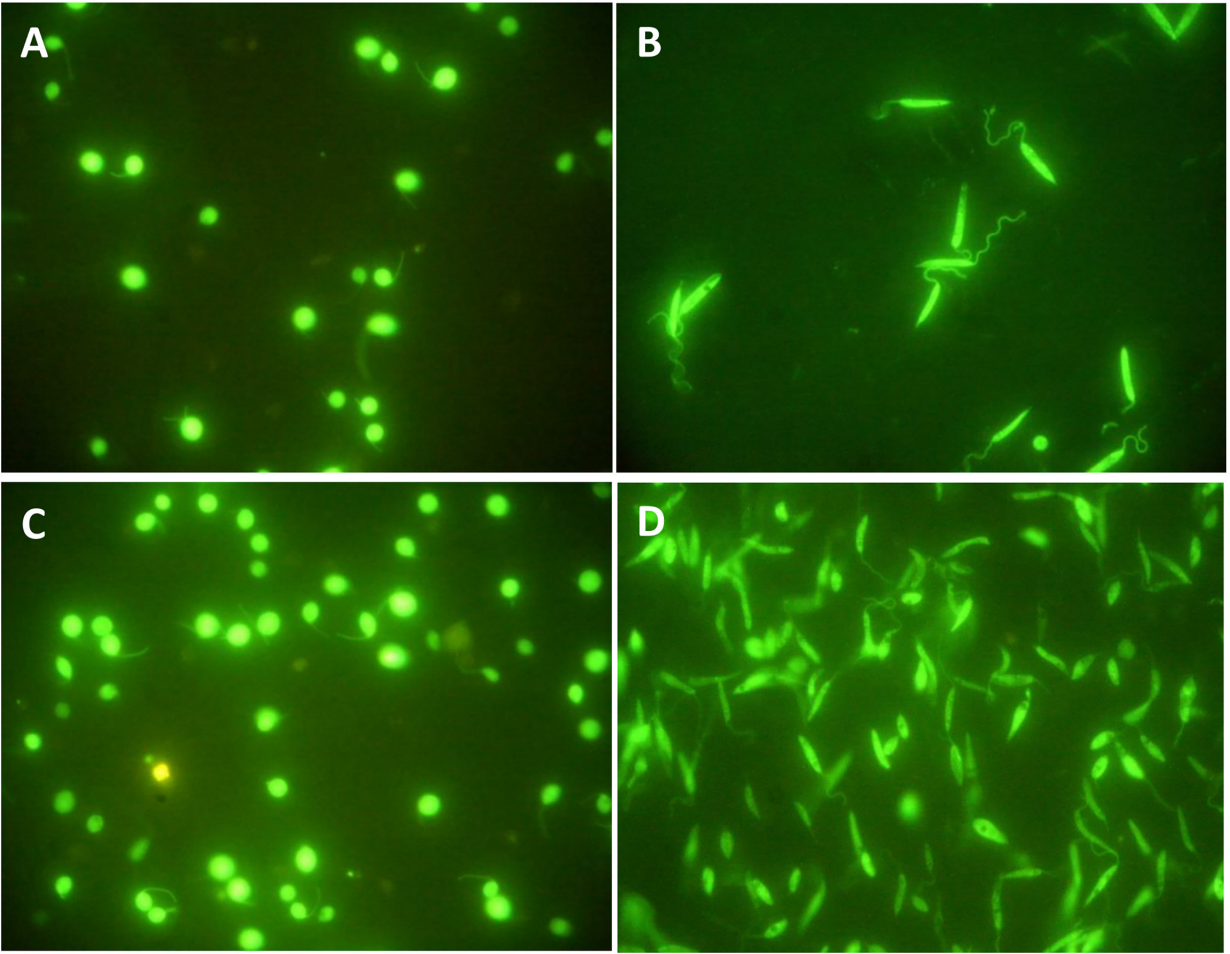
**(A)** *L. tarentolae* ^GFP+^ and **(B)** *L. major* ^GFP+^ on the fourth day of cultivation and before any manipulation; both species express GFP. **(C)** *L. tarentolae* ^GFP+^ after 4 h of incubation at 26°C. **(D)** *L. major* ^GFP+^ after 4 h of incubation at 37°C. No difference in morphology was observed. All images were taken using a fluorescence microscope at ×100 magnification (Nikon ECLIPSE E200, Japan).

In order to morphologically assess the culture conditions on the studied parasites, GFP-producing species were used in this study, and their morphology was observed before and after 4 h of incubation in serum-free media at 26° and 37°C for *L. tarentolae* ^GFP+^ and *L. major* ^GFP+^, respectively. These temperatures were chosen considering the internal temperature of each species’ hosts (Barbosa et al., 2018) (human and *T. annularis*, respectively). No morphological changes were observed under a fluorescent microscope (**Figure 1**). In addition, the percent reduction of Resazurin solution after 4 h of incubation in serum-free media showed high levels of the parasite’s viability in both species (**Supplementary Figure S2**). For all the viability tests, 10^7^ parasites per well were subjected to Resazurin solution since two individual experiments on *L. tarentolae* ^GFP+^ and *L. major* ^GFP+^ showed that the best concentration of parasites is 10^7^ parasites per well for such viability tests (**Supplementary Figure S3**).

### Capturing the EV secretion event by FE-SEM

**Figure 2:**
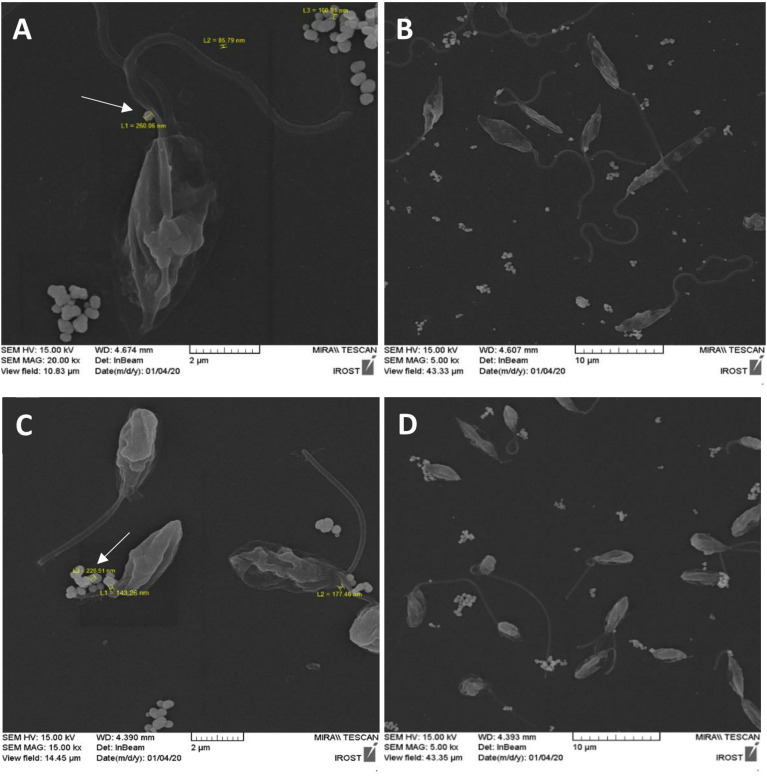
FE-SEM image after 4 h of culture in serum-free medium clearly showing the production of extracellular vesicles (EVs) by both species. **(A)** *L. major* ^GFP+^ at 37°C with 2-µm scale bar. The arrow indicates an EV produced by *L. major* ^GFP+^ with a diameter of 260 nm. **(B)** *L. major* ^GFP+^ with 10-µm scale bar. **(C)** *L. tarentolae* ^GFP+^ at 26°C with 2-µm scale bar. The arrow indicates an EV produced by *L. tarentolae* ^GFP+^ with a diameter of 226 nm. **(D)** *L. tarentolae* ^GFP+^ with 10-µm scale bar.

One piece of evidence that proves the production of extracellular vesicles by *L. tarentolae* is their pictures after incubation in a serum-free culture medium. Since there are no vesicles in the medium by default due to the lack of FBS, EVs would be observed in the culture medium only if the parasites produced them. It should be noted that *L. major* ^GFP+^, cultivated in similar conditions, has been used as a positive control. The negative control of this test was *L. major* ^GFP+^ and *L. tarentolae* ^GFP+^ cultivated in the same culture condition, with the difference that both parasites were killed and fixed using Thiomresol (Merck, Germany) at the beginning of the 4 h incubation to make sure that the aforesaid EVs are released only by parasites (**Supplementary Figure S4**). Among the most typical images published of *Leishmania* EVs are images that show a viable and morphologically intact parasite during the production of vesicles around the flagellum (Silverman and Reiner, 2012; Barbosa et al., 2018; Atayde et al., 2019; Dong and Olivier, 2019; Nogueira et al., 2020). The SEM images of these two species demonstrate the production of EVs by the parasites (**Figure 2**). In **Figure 2A**, the scale bar represents 2 µm, while the arrow indicates an EV produced by *L. major* ^GFP+^ with a diameter of 260 nm. In **Figure 2B**, the same parasite (*L. major* ^GFP+^) is shown at a lower magnification (the scale bar is 10 µm). In **Figure 2C**, the scale bar represents 2 µm, and the arrow indicates an EV produced by *L. tarentolae* ^GFP+^ with a diameter of 226 nm, while in **Figure 2D**, the same parasite (*L. tarentolae* ^GFP+^) is shown at a lower magnification (the scale bar is 10 µm).

### The size and the concentration of tEV and mEV were measured using NTA and BCA

**Figure 3:**
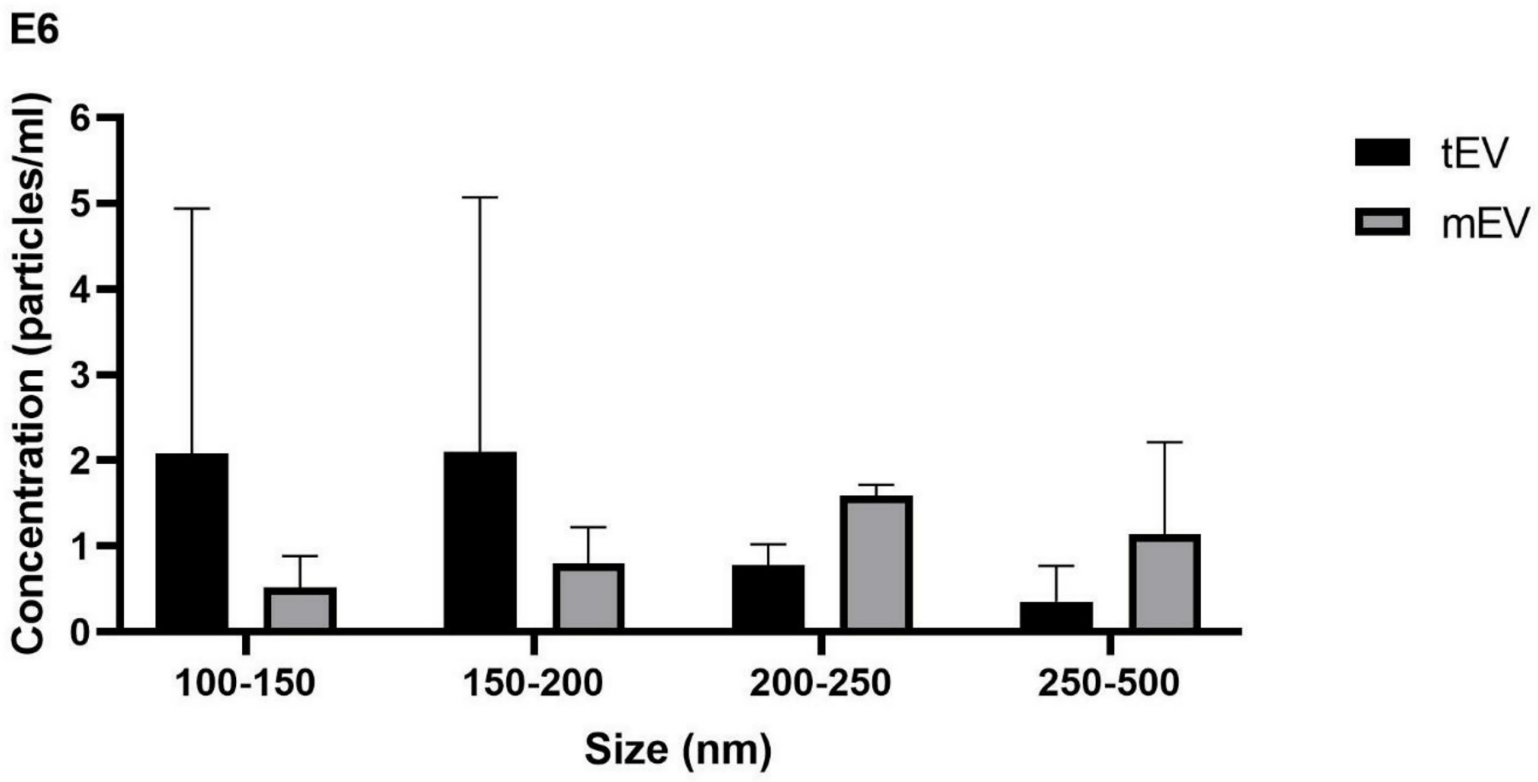
Size and concentration demonstrated by nanoparticle tracking analysis (NTA) showing that tEV released from *L. tarentolae* ^GFP+^ and mEV derived from *L. major* ^GFP+^ contain 5.92 × 10^8^ and 5.45 × 10^8^ particles per milliliter, respectively. The bar chart depicts a comparison between tEV and mEV in terms of their concentration in different size intervals based on NTA. Most particles (D90) in both species were below 500 nm, and one-way ANOVA showed no significant differences between tEV and mEV within the size ranges considered. This graph represents the approximate concentration (particles/milliliter) in two individual rounds of the NTA assay.

As stated by two independent measurements by NTA, tEV released from *L. tarentolae* ^GFP+^ contains 5.92 × 10^8^ particles per milliliter, and BCA revealed that tEV includes 20 µg protein per milliliter in total. For mEV released from *L. major* ^GFP+^, NTA showed 5.45 × 10^8^ particles per milliliter, and BCA showed that mEV includes 23 µg protein per milliliter in total. As previously described, particle number per protein ratio was calculated, which was 29.6 × 10^6^ and 23.6 × 10^6^ particles/µg for tEV and mEV, respectively. In **Figure 3**, the size distribution of particles is divided into four intervals (100–150, 150–200, 200–250, and 250–500 nm) to specify and draw a comparison between tEV with mEV in order to determine the particles’ size distribution within each size range. The bar graph has been drawn based on the distribution of approximate concentrations and size of the isolated EVs. In accordance with ordinary one-way ANOVA, no significant differences were detected in the given size intervals between mEV and tEV. However, the graph shows a tendency to smaller particles in tEVs compared with mEVs. Furthermore, in both species, the size of 90% of particles (D90) was below 500 nm (**Figure 3**).

### Both tEV and mEV contain GP63 and GFP

One of the most important proofs for the presence of extracellular vesicles in a solution is the presence of its specific markers, according to the MISEV2018 (Théry et al., 2018). The existence of EVs in a solution can be confirmed by proving the presence of a transmembrane protein or a GPI-anchored protein (here GP63) and a cytosolic protein (here GFP) in solution.

**Figure 4:**
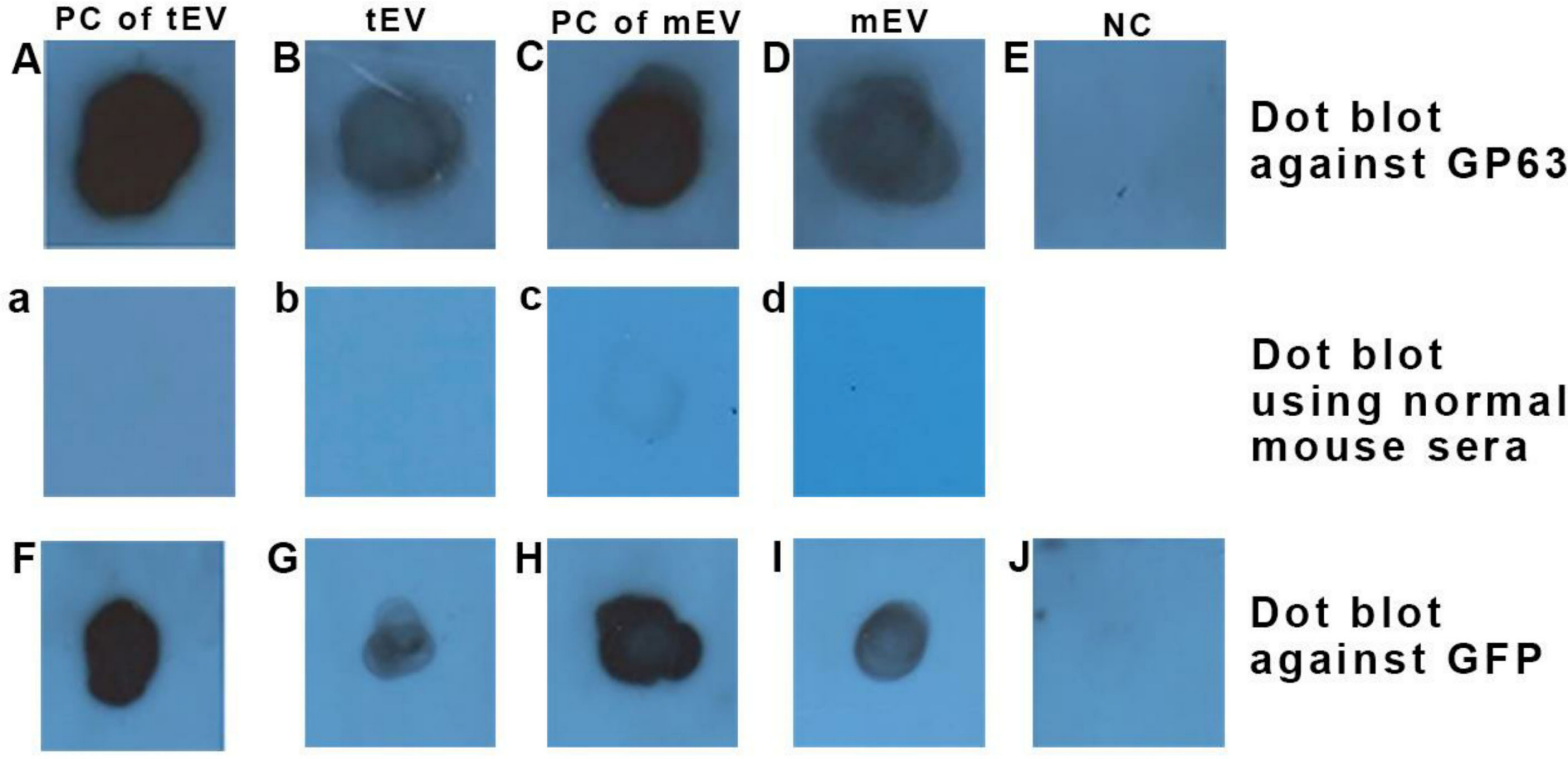
Dot blot using anti-GP63 (upper row) confirmed the presence of this GPI-anchored extracellular vesicle (EV) marker in EVs of both species and dot blot using anti-GFP (lower row) confirmed the presence of this cytosolic EV marker within EVs of both species. **(A, F)** *L. tarentolae* ^GFP+^ extract as a positive control for tEV (35 µg protein in total). **(B, G)** *L. tarentolae* ^GFP+^ EVs or tEV (1 µg in total). **(C, H)** *L. major* ^GFP+^ extract as a positive control for mEV (35 µg in total). **(D, I)** *L. major* ^GFP+^ EVs or mEV (1 µg in total). **(E, J)** Negative control (phosphate-buffered saline). The middle row depicts a separate dot blot using normal mouse sera as the primary antibody, which serves as a negative control. **(a)** *L. tarentolae* ^GFP+^ extract (same as **A, F**). **(b)** *L. tarentolae* ^GFP+^ EVs or tEV (similar to **B, G**). **(c)** *L. major* ^GFP+^ extract (same as **C, H**). **(d)** *L. major* ^GFP+^ EVs or mEV (similar to **D, I**). PC, positive control; tEV, *L. tarentolae* EV; mEV, *L. major* EV; NC, negative control.

The dot blot analysis detected the expression of GP63, which is shown in **Figure 4** (upper row), where in **Figure 4A**, the positive reactivity of *L. tarentolae* ^GFP+^ extract (as a positive control for tEV) is shown, and in **Figure 4B**, the positive reactivity of *L. tarentolae* ^GFP+^ qEV column’s product (tEV) is depicted. In addition, in **Figure 4C**, the positive reactivity of *L. major* ^GFP+^ extract (as a positive control for mEV) is shown, while in **Figure 4D**, the positive reactivity of *L. major* ^GFP+^ qEV column’s product (mEV) is depicted. **Figure 4E** shows the negative reactivity of PBS as the negative control.

The dot blot analysis also detected the expression of GFP as shown in **Figure 4** (lower row), wherein **Figure 4F** shows that the *L. tarentolae* ^GFP+^ extract (as a positive control for tEV) has a positive reactivity to anti-GFP. In **Figure 4G**, the positive reactivity of *L. tarentolae* ^GFP+^ qEV column’s product (tEV) is shown. **Figure 4H** shows the positive reactivity of *L. major* ^GFP+^ extract (as a positive control for mEV), and in **Figure 4I**, the positive reactivity of *L. major* ^GFP+^ qEV column’s product (mEV) is demonstrated. Finally, in **Figure 4J**, the negative reactivity of PBS (as the test’s negative control) is shown.

In the middle row, non-related sera (normal mouse sera) were used as the primary antibody in order to rule out any chances of the presence of endogenous peroxidase activity. The panels a, b, c, and d, which represent the same samples with **Figures 4A–D**, showed no positive reactivity with the aforementioned antibody.

### EV-treated macrophages contain GP63

**Figure 5:**
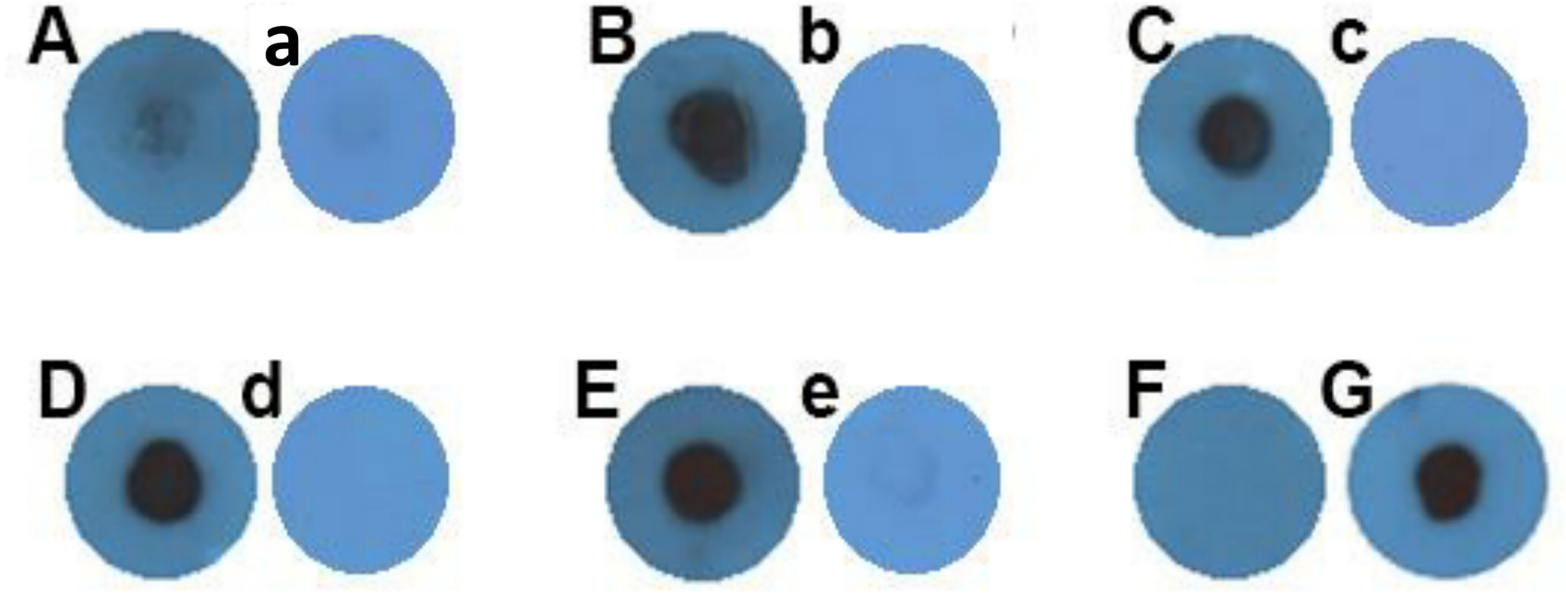
Dot blot against GP63 on THP-1 macrophages treated with *Leishmania* EVs. **(A)** Negative control (macrophage without extracellular vesicle treatment). **(B)** THP-1 macrophages treated with tEV. **(C)** THP-1 macrophages treated with mEV. **(D)** THP-1 macrophages infected only with *L. major* ^GFP+^. **(E)** *L. major* ^GFP+^ extract. The concentration of all samples so far was 35 µg in total. **(F)** Negative control (phosphate-buffered saline). **(G)** β-actin positive control 35 µg THP-1, same sample as **(A)**. All of the panels labeled with lowercase letters (**a–e**) are representative of another individual experiment on which normal mouse sera were used as the primary antibody with the same samples as those in each of the uppercase-labeled panels.

GP63 protein is expressed only in parasites and not in THP-1 cells (Isnard et al., 2012); therefore, the existence of this protein in macrophages treated with *Leishmania* EVs, in addition to proving the ability of origination of these vesicles by *L. tarentolae*, can also prove the successful uptake of these vesicles by the macrophages. In this regard, the extract of THP-1 macrophages which were treated with these vesicles and washed after 24 h, was subjected to dot blot analysis to detect the presence or absence of this protein inside them. In comparison to negative controls, macrophages treated with tEV or mEV contain GP63 in their extract (**Figure 5**). **Figure 5A** indicates the negative control, which is THP-1 macrophages without any EV treatment. **Figures 5B** and **C** represent THP-1 macrophages treated with tEV and mEV, respectively. **Figure 5D** corresponds to THP-1 macrophages infected only with *L. major* ^GFP+^, and **Figure 5E** represents *L. major* ^GFP+^ lysate. In **Figure 5G**, β-actin served as the primary antibody as a positive control for cell labeling. **Figures 5E** and **G** were assumed as positive controls. About 35 µg protein was used for each blot. **Figure 5F** was PBS, which served as the test’s negative control. Furthermore, there is a negative control for each sample marked with lowercase letters (a–e), which shows the samples’ reactivity with normal mouse sera as the primary antibody.

### Treatment with tEVs induced cytokines in THP-1 macrophages

**Figure 6:**
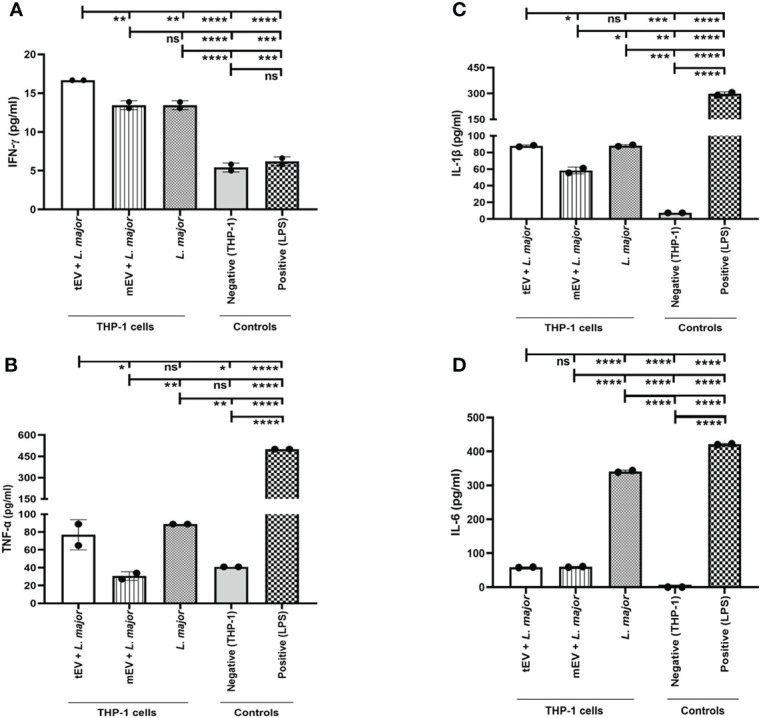
Cytokine assay on extracellular vesicle-treated and / or -infected THP-1 macrophages. Here different groups were included as mentioned in the result section (Treatment with tEVs induced cytokines in THP-1 macrophages). **(A)** IFN-γ production in 72 h showed a significant (**p-value = 0.0076) increase in the main test group (tEV + L. major) compared with both mEV+*L. major* and *L. major* groups. In addition, the statistical analysis between the main test group and other control groups (positive and negative control) showed a significant (****p-value < 0.0001) increase. The detection limit was 9–600 pg per milliliter. **(B)** TNF-α production in 24 h demonstrated a significant (*p-value = 0.0107) increase between the main test group and THP-1 macrophages which were treated with mEV prior to infection (mEV + *L. major*), whereas no significant differences were seen between the test group and the macrophages that were only infected with *L. major*. The detection limit was 15–1,000 pg/ml. **(C)** IL-1β production in 72 h showed a significant (*p-value = 0.0177) increase in the main test group compared with the mEV + *L. major* group, whereas no significant differences were detected between the test and the *L. major* group. Furthermore, the IL-1β levels were significantly increased with *p*-value = 0.0002 compared with the negative control group, but these levels were significantly reduced with *p*-value <0.0001 in comparison with the positive control group. The detection limit was 3–250 pg/ml. **(D)** IL-6 production in 48 h showed a significant (****p-value < 0.0001) decrease in the test group in comparison with the *L. major* group, while no significant differences were detected between the test and the mEV + *L. major* group. In a comparison between the test group and both control groups, the differences with *p*-value <0.0001 were significant, where the production of this cytokine was increased and decreased compared with the negative and positive control groups, respectively. The detection limit was 9–600 pg/ml. All of the results were derived from two individual experiments. The asterisk indicates the significant difference between the values of the indicated groups as determined by one-way ANOVA followed by Tukey’s test (*p < 0.05, **p < 0.01, ***p < 0.001, ****p < 0.0001, and ns, non-significant).

Another indicator of the effects of EVs on human macrophages is the production of inflammatory or pro-inflammatory cytokines by these cells when treated with EVs and infected by a pathogenic species such as *L. major* ^GFP+^. To achieve this, the supernatants of THP-1 macrophages treated with EVs and then infected with parasites were collected after 24, 48, and 72 h and used to measure the concentrations of secreted TNF-α, IFN-γ, IL-1β, and IL-6. To reach a better assessment of the effects of tEV on cytokine response, three different groups were designed; one of those was THP-1 macrophages which were only infected with *L. major* without any EV treatment, while another group was THP-1 macrophages treated with mEV prior to infection with *L. major* ^GFP+^. Both of these groups served as control groups. The last group was THP-1 macrophages treated with tEV before infection with *L. major* ^GFP+^. Furthermore, this experiment had two more control groups, one of which was the LPS control group as a positive control (due to its ability to increase the above-mentioned cytokines) (Meng and Lowell, 1997) that was THP-1 macrophages treated only with LPS, and the other control group was THP-1 macrophages without any treatment or infection, which serves as the experiment’s negative control. The results demonstrated in **Figure 6** revealed that tEV increased IFN-γ compared to both designated groups (mEV + *L. major* ^GFP+^ and *L. major* ^GFP+^). This increase was significant by (**p-value = 0.0076). However, the differences between tEV+ *L. major* and both control groups were (****p-value < 0.0001) significant (**Figure 6A**). In addition, tEV significantly increased the TNF-α levels compared to mEV + *L. major* (*p-value = 0.0107) and the negative control group (*p-value = 0.0305). On the other hand, TNF-α production was significantly decreased (****p-value < 0.0001) compared with the main test group and the group that had been treated with LPS (**Figure 6B**). In regard to IL-1β (**Figure 6C**), no significant difference was detected compared with the THP-1 macrophages, which were only infected with *L. major*, but a significant increase (*p-value = 0.0177) was observed in comparison with the THP-1 cells that were treated with mEV before infection. Interestingly, we observed a significant decrease (****p-value < 0.0001) in IL-6 production in both EV-treated groups compared with THP-1 macrophages which were only infected with *L. major*, but no significant differences were observed between the two EV-treated groups (**Figure 6D**), which can be due to the virulence factors associated with *Leishmania* EVs. However, the IL-6 levels of both EV-treated groups were significantly increased (****p-value < 0.0001) compared to the negative control and significantly decreased (****p-value < 0.0001) in comparison with the positive control or LPS group. Lastly, the effect of tEV alone on all of the assessed cytokines was measured, but they failed to exert any statistically significant effect on cytokine production (data not shown).

### tEV decreased the infection rate in THP-1 macrophages

**Figure 7:**
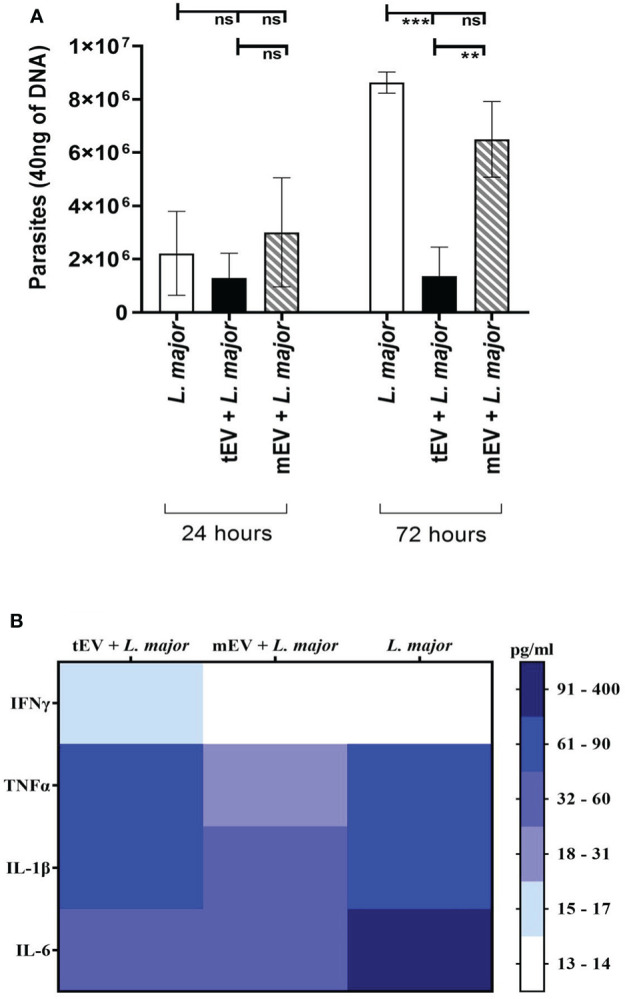
**(A)** The number of parasites present in 40 ng DNA of THP-1 macrophage after 24 and 72 h. In the two experimental groups, the macrophages were first treated with tEV or mEV for 24 h, and to evaluate their effect on THP-1 human macrophages, they were infected with the pathogenic *L. major* for 6 h, while the parasite burden was assessed using the genome extracted from them with *Leishmania*-specific RV1/RV2 primer. According to one-way ANOVA along with post-hoc Tukey’s test, the statistical analysis of three individual experiments, in the 72-h results, showed a significant difference (***p-value = 0.0003) between the control group—which was only infected with L. major—and the test group—which was treated with tEV before infection—which was significant (***p-value = 0.0003), furthermore, between the test group and the reference group that was treated with mEV before infection had a significant difference (**p-value = 0.0056). In 72 h, the differences between the control group and the reference group and also between all 24-h groups were not significant (*p < 0.05, **p < 0.01, ***p < 0.001, and ns, non-significant). **(B)** Cumulative comparison of the cytokine results (**Figure 6**) shown as a heat map for further comparison between tEV + *L. major* group with other groups in all studied cytokines.

One of the main questions of this study is to find the effects of tEV on THP-1 macrophages. Since one hypothesis of this study is to use these EVs for therapeutic applications, it is very important to make sure that these EVs lack any adverse effects on human immune cells. In this regard, one of the best indicators of these effects is the propagation of parasites within EV-treated THP-1 macrophages after infection with *L. major* as a pathogenic parasite. For this purpose, the genomic DNA extracted from different groups of THP-1 cells was subjected to real-time PCR using RV1/RV2 primers, which is specific to *Leishmania*. For a better understanding of these effects, three different groups of THP-1 cells were assigned for real-time PCR; the control group represents THP-1 macrophages that were only infected with *L. major* without any EV treatment (THP-1 + *L. major*), the reference group represents THP-1 macrophages that were treated with mEV prior to infection with *L. major* (THP-1 + mEV + *L. major*), and the test group represents THP-1 macrophages treated with tEV before infection with *L. major* (THP-1 + tEV + *L. major*). The results demonstrated in **Figure 7A** do not show a significant difference between the control group and the reference group. However, in the test group, there was a significant reduction in parasitic burden compared to both the reference and the control groups after 72 hours. This clearly indicates the relative control of parasite propagation in THP-1 macrophages by *L. tarentolae*’s extracellular vesicles after infection with *L. major*. In **Figure 7B**, all of the results from the cytokine measurements have been summarized as a heat map which only includes the same THP-1 macrophage groups that were subjected to real-time PCR in order to draw a comparison between tEV + *L. major* group and mEV + *L. major* along with the group which was without any EV treatment (*L. major*). By providing this comparison, it is evidently clear that, unlike mEV, tEV managed to increase IFN-γ, TNF-α, and IL-1β while decreasing IL-6 and parasite burden.

## Discussion

Nowadays, extracellular vesicles have drawn much attention from scientists in different fields due to their enormous functional capacity and remarkable potential applications. In pathogenic *Leishmania*, such as *L. major*, it is sufficiently established that EVs play an important role at the primary stages of infection and in promoting disease progression. *L. tarentolae*, on the other hand, is a non-pathogenic parasite that is proposed as a promising approach for vaccine development by many studies. However, no study so far has provided evidence for the production and secretion of EVs by *L. tarentolae*.

As the first step of the present study, the ability of *L. tarentolae* in producing EVs was demonstrated. In the second step, the function and the effects of these EVs on THP-1 macrophages were assessed. Macrophages are considered as the main host of *Leishmania* parasites in humans (Al-Kamel, 2017); therefore, examining the effects of these vesicles on macrophages is a fundamental step towards understanding EVs’ effects in disease progression and control.

Parasite adaptation to different hosts may be reflected in EV secretion and components. During the life cycle of *Leishmania*, it faces several inimical conditions not only in the sand fly (approximately 26°C) but also in the vertebrate host (37°C) (Barbosa et al., 2018). As mentioned earlier, there are no reports on non-pathogenic *Leishmania* EVs so far, and studies on pathogenic *Leishmania* EVs have confirmed the regulatory impacts of such EVs on the immune system in order to survive inside their hosts. These specific regulatory effects are induced by infection-like temperatures (Castelli et al., 2019). In this regard, EVs of *L. major* and *L. tarentolae* were studied based on their host temperature (human: 37°C, *T. annularis*: 26°C) in order to have the best simulation of their infection process and to have a better illustration of their immune regulatory effects.

Many studies, using FE-SEM, have captured viable and morphologically intact *Leishmania* during the production of vesicles near the flagellum pocket (Silverman and Reiner, 2012; Barbosa et al., 2018; Atayde et al., 2019; Dong and Olivier, 2019; Nogueira et al., 2020). Interestingly, when we fixed the parasites using Thiomresol, no vesicular structure was seen in the FE-SEM images (**Supplementary Figure S4**), while there were numerous vesicles around viable parasites that have not been treated with Thiomresol (**Figure 2**).

Immunoblotting is an integral part of almost every study on EVs. Most of the studies have done this using Western blotting, but none of the studies on *Leishmania* EVs has the same extraction method as the one used in this study. In a study on EVs in human plasma, several methods of purification of extracellular vesicles were used, one of which was similar to this study. The differences between different extraction and purification methods and their effect on the properties of the obtained vesicles are well illustrated. Although using qEV columns provided the highest ratio of particles per milliliter of protein, due to the high purity, the total number of particles and the total amount of protein obtained from this column were very low (Lobb et al., 2015). As a result, it may not be possible to see a clear band using the Western blot technique. For this reason, we used the dot blot technique here since this study aims to prove the presence of EVs in *L. tarentolae*; on the other hand, the EV extraction method used in this study has the highest purity (removes 97% of impurities). According to MISEV2018 (Théry et al., 2018), proving the presence of a GPI-anchored protein and a cytosolic protein is required. As previously reported, *L. tarentolae* possesses an approximately 63-kDa protein that is antigenically cross-reactive with *L. major* GP63, but its lack of protease activity is considered a major cause of non-pathogenicity of *L. tarentolae* for mammalian cells (Campbell et al., 1992; Contreras et al., 2010; Raymond et al., 2012; Valdivia et al., 2015). Therefore, we selected GP63 as the GPI-anchored marker for both species. The positive reactivity of both cytosolic and GPI-anchored markers using dot blot was another proof for the ability of *L. tarentolae* to produce EVs. Moreover, detecting GP63, a protein that is not expressed by human macrophages, inside the tEV-treated macrophages showed a successful uptake of them by these macrophages along with proving the ability of *L. tarentolae* in EV production—an ability of which there has not been any report before—yet further proteomic analysis is required in EVs derived from both species and EVs isolated from *Leishmania*-infected human macrophages.

Particle size and concentration are among the most important indicators of purity of an EV sample. There are many studies reporting NTA for *Leishmania* EVs, and most of them have approximately similar size and concentration as reported in the present study (Lambertz et al., 2015; Barbosa et al., 2018; Douanne et al., 2020; Gioseffi et al., 2020; Nogueira et al., 2020; Heirwegh et al., 2021). One of the most similar studies on *Leishmania* EVs in terms of NTA results is done by Olivier *et al.* and showed similar concentration and approximately similar size of EVs (Atayde et al., 2019). The only difference with this study is finding the most particles below 300 nm, while in this study, most of the particles (D90) are below 500 nm. In conclusion, using NTA, we discovered that most EVs in *L. tarentolae* ^GFP+^ and *L. major* ^GFP+^ were in an approximately same size and protein concentration, and the particle number per protein ratio was relatively similar between these two studied species. Nevertheless, tEVs possessed a larger population in smaller size distributions compared with mEVs without having any statistically significant difference. In general, NTA and BCA showed similar characteristics between the EVs produced by *L. major* ^GFP+^ and *L. tarentolae* ^GFP+^, which is another proof for the secretion of EVs by *L. tarentolae*.

In contrast with several studies showing the adverse effects of EV-depleted FBS (EFBS) on growth, differentiation, and other factors based on the studied cell type (Sun et al., 2014; Eitan et al., 2015; Li et al., 2015; Angelini et al., 2016; Aswad et al., 2016; Gudbergsson et al., 2016; Cavallari et al., 2017; Gimona et al., 2017), we have found no significant effect on THP-1 propagation, at least within the first 96 h of cultivation as done in this study. On the other hand, in all the experiments in this study, macrophages were cultured for a maximum of 72 h, during which the survival rate of THP-1 macrophages was close to or equal to 100% (**Supplementary Figure S5**).

Studies examining the interactions between parasites and hosts have shown that pathogenic parasites in humans usually secrete EVs in response to environmental changes to facilitate the conditions for their pathogenesis (Torrecilhas et al., 2020). In this regard, it was shown that EVs can affect the immune system and transmit various virulence factors to target cells or related cells so that they can manipulate their life cycle by turning on or off the signaling pathways involved in cell death (Saljoughian et al., 2013; Castelli et al., 2019). Furthermore, they can alter the transcription process of target cells, suppress immune responses, and increase disease severity or increase the parasite burden by induction of Th2 response (Barbosa et al., 2018). In general, *in vitro* and *in vivo* studies suggest that the abundance of *Leishmania* virulence factors in their EVs is a major facilitator of the infection process and the development of other detrimental effects related to leishmaniasis (Dong and Olivier, 2019). Interferon gamma plays an important role in the development and control of *Leishmania* infections. IFN-γ and some other cytokines and chemokines can influence the course of infection in infectious diseases (*e*.*g*., protozoan parasites). *Leishmania* parasites elaborate factors that modulate activities induced by IFN-γ (Kima and Soong, 2013). Stimulated with microbial products, IFN-γ, or IL-12, macrophages are able to secrete considerable amounts of IFN-γ that show their impacts on linking innate and adaptive immunity (Gessani and Belardelli, 1998; Kraaij et al., 2014). In addition, TNF-α is a crucial cytokine in the inflammatory cascade by activating the Th1 immune response. This particular cytokine in leishmaniasis is so important that TNF-α blocker-based therapy seems to be associated with a higher risk of infections, at times with a worse outcome (Bosch-Nicolau et al., 2019). IL-1β, on the other hand, which is produced as a precursor protein by active macrophages, is one of the most important factors in the inflammatory response in macrophages (Kaneko et al., 2019). The latest studied cytokine, IL-6, which is a pleiotropic cytokine, has been associated with the induction of acute-phase protein synthesis and production of anti-inflammatory proteins and favors the development of Th2 response, which suppresses the activation and antimicrobial effect of macrophages (Dayakar et al., 2019). *L. tarentolae* has the potential for use as a safe live vector in vaccination (Saljoughian et al., 2013), and we hypothesized similarly about its EVs. Numerous studies have shown that EVs derived from pathogenic *Leishmania* have a quenching effect on such inflammatory cytokines in order to evade the human immune system (Torrecilhas et al., 2020), while our results showed a significant increase in IFN-γ and no significant difference in IL-1β between THP-1 macrophages that had been treated with tEV prior to *Leishmania* infection and the macrophages that were only infected with *L. major* without any pretreatment. In addition, in a comparison between tEV-treated THP-1 macrophages and mEV-treated macrophages, the levels of IFN-γ, TNF-α, and IL-1β were significantly increased. In summary, we showed that the pre-treatment of tEVs somehow warned the THP-1 macrophages by increasing IFN-γ, TNF-α, and IL-1β and by decreasing IL-6 simultaneously, and a significant decrease was observed in parasite burden when tEV-treated macrophages were infected with a pathogenic *Leishmania* parasite. Although there is a significant difference between tEV+ *L. major* with mEV+ *L. major* group and *L. major* group, the level of IFN-γ in **Figure 6A** is not high enough (Baxter et al., 2020) to rest assured about a meaningful correlation between the increase of this cytokine and the decrease seen in parasite burden. Consequently, it cannot be concluded that a rise in IFN-γ was the chief reason that activated the microbicidal mechanisms. As mentioned earlier, no research has been manifested on the secretion and characteristics of EVs by non-pathogenic *L. tarentolae*, and herein we provided the first clear evidence of reducing the infection rate in THP-1 macrophages by *L. tarentolae*’s EVs, among other demonstrated physical and chemical hallmarks of tEVs.

In conclusion, more investigations through the characteristics of tEVs are of significant importance since they have a perspective to serve as a new pathway for drug delivery, among other applications, in the near future. It is crucial to set up a comprehensive understanding of tEVs with respect to protein content, molecular makeup, cytokine alterations, and *in vivo* studies in more detail.

## Data availability statement

The original contributions presented in the study are included in the article/**Supplementary Material**. Further inquiries can be directed to the corresponding authors.

## Author contributions

Conceptualization: SR, AM, and FS. Data curation: MS. Formal analysis: MS. Funding acquisition: SR. Investigation: MS, HS, YT, ML, and SH. Methodology: MS, SR, AT, and FS.

Project administration: SR. Resources: SR. Supervision, validation, and visualization: SR and AT. Writing—original draft: MS. Writing—review and editing: SR, AT, AM, FS, and MB. All authors contributed to the article and approved the submitted version.

## Funding

This project was supported by two grants from the National Institute for Medical Research Development (NIMAD, 982982), Tehran, Iran, and the European Union’s Horizon 2020 Research and Innovation Program under the Marie Skłodowska Curie Actions (grant number 778298).

## Acknowledgments

The authors are grateful to Dr. Ali M. Harandi (University of Gothenburg), Dr. Behrouz Vaziri and Dr. Fatemeh Torkashvand (Biotechnology Research Center, Pasteur Institute of Iran), Dr. Patricia Xander Batista (Federal University of São Paulo), and Dr. Farnaz Zahedifard, Dr. Tahereh Taheri, and Dr. Negar Seyed (Department of Immunotherapy and *Leishmania* Vaccine Research, Pasteur Institute of Iran) who provided insight and expertise that greatly assisted the research. We would also like to show our gratitude to Dr. Farzaneh Barkhordari (Pasteur Institute of Iran) for kindly providing anti-GP63 antibody, and to Dr. Reza Saghiri, Dr. Dariush Norouzian, and Dr. Mohammad Barati (Pasteur Institute of Iran) for their facilities on providing different materials and equipment throughout this project. In addition, we acknowledge Mr. Shahram Gholam-Alizadeh (Department of Immunotherapy and *Leishmania* Vaccine Research, Pasteur Institute of Iran) for his technical help and artworks.

## Conflict of interest

The authors declare that the research was conducted in the absence of any commercial or financial relationships that could be construed as a potential conflict of interest.

## Publisher’s note

All claims expressed in this article are solely those of the authors and do not necessarily represent those of their affiliated organizations, or those of the publisher, the editors and the reviewers. Any product that may be evaluated in this article, or claim that may be made by its manufacturer, is not guaranteed or endorsed by the publisher.

## References

[B1] Available at: https://www.who.int/en/news-room/factsheets/detail/leishmaniasis.

[B2] Abdossamadi Z. Taheri T. Seyed N. Montakhab-Yeganeh H. Zahedifard F. Taslimi Y.. (2017). Live leishmania tarentolae secreting HNP1 as an immunotherapeutic tool against leishmania infection in BALB/c mice. Immunotherapy 9 (13), 1089–1102. doi: 10.2217/imt-2017-0076 29032739

[B3] Al-Kamel M. A. (2017). Stigmata in cutaneous leishmaniasis: Historical and new evidence-based concepts. Our Dermatol. Online/Nasza Dermatol Online 8 (1), 81–90. doi: 10.7241/ourd.20171.21

[B4] Alvar J. Vélez I. D. Bern C. Herrero M. Desjeux P. Cano J.. (2012). Leishmaniasis worldwide and global estimates of its incidence. PloS One 7 (5), e35671. doi: 10.1371/journal.pone.0035671 22693548 PMC3365071

[B5] Angelini F. Ionta V. Rossi F. Miraldi F. Messina E. Giacomello A. (2016). Foetal bovine serum-derived exosomes affect yield and phenotype of human cardiac progenitor cell culture. BioImpacts: BI 6 (1), 15–24. doi: 10.15171/bi.2016.03 27340620 PMC4916547

[B6] Aswad H. Jalabert A. Rome S. (2016). Depleting extracellular vesicles from fetal bovine serum alters proliferation and differentiation of skeletal muscle cells *in vitro* . BMC Biotechnol. 16 (1), 1–12. doi: 10.1186/s12896-016-0262-0 27038912 PMC4818850

[B7] Atayde V. D. Aslan H. Townsend S. Hassani K. Kamhawi S. Olivier M. (2015). Exosome secretion by the parasitic protozoan leishmania within the sand fly midgut. Cell Rep. 13 (5), 957–967. doi: 10.1016/j.celrep.2015.09.058 26565909 PMC4644496

[B8] Atayde V. D. da Silva Lira Filho A. Chaparro V. Zimmermann A. Martel C. Jaramillo M.. (2019). Exploitation of the leishmania exosomal pathway by leishmania RNA virus 1. Nat. Microbiol. 4 (4), 714–723. doi: 10.1038/s41564-018-0352-y 30692670

[B9] Atayde V. D. Hassani K. da Silva Lira Filho A. Borges A. R. Adhikari A. Martel C.. (2016). Leishmania exosomes and other virulence factors: impact on innate immune response and macrophage functions. Cell. Immunol. 309, 7–18. doi: 10.1016/j.cellimm.2016.07.013 27499212

[B10] Barbosa F. M. C. Dupin T. V. Toledo M. D. S. Reis N. F. D. C. Ribeiro K. Cronemberger-Andrade A.. (2018). Extracellular vesicles released by leishmania (Leishmania) amazonensis promote disease progression and induce the production of different cytokines in macrophages and b-1 cells. Front. Microbiol. 9, 3056. doi: 10.3389/fmicb.2018.03056 30627118 PMC6309564

[B11] Basile G. Peticca M. (2009). Recombinant protein expression in leishmania tarentolae. Mol. Biotechnol. 43 (3), 273. doi: 10.1007/s12033-009-9213-5 19779853

[B12] Baxter E. Graham A. Re N. Carr I. Robinson J. Mackie S.. (2020). Standardized protocols for differentiation of THP-1 cells to macrophages with distinct m (IFNγ+ LPS), m (IL-4) and m (IL-10) phenotypes. J. Immunol. Methods 478, 112721. doi: 10.1016/j.jim.2019.112721 32033786

[B13] Besteiro S. Williams R. A. Coombs G. H. Mottram J. C. (2007). Protein turnover and differentiation in leishmania. Int. J. Parasitol 37 (10), 1063–1075. doi: 10.1016/j.ijpara.2007.03.008 17493624 PMC2244715

[B14] Bolhassani A. Taheri T. Taslimi Y. Zamanilui S. Zahedifard F. Seyed N.. (2011). Fluorescent leishmania species: development of stable GFP expression and its application for *in vitro* and *in vivo* studies. Exp. Parasitol 127 (3), 637–645. doi: 10.1016/j.exppara.2010.12.006 21187086

[B15] Bosch-Nicolau P. Ubals M. Salvador F. Sánchez-Montalvá A. Aparicio G. Erra A.. (2019). Leishmaniasis and tumor necrosis factor alpha antagonists in the Mediterranean basin. A Switch Clin. Expression PloS Negl. Trop. Diseases 13 (8), e0007708. doi: 10.1371/journal.pntd.0007708 PMC6742442 31469834

[B16] Breton M. Tremblay M. J. Ouellette M. Papadopoulou B. (2005). Live nonpathogenic parasitic vector as a candidate vaccine against visceral leishmaniasis. Infect Immunity 73 (10), 6372–6382. doi: 10.1128/IAI.73.10.6372-6382.2005 16177308 PMC1230936

[B17] Campbell D. A. Kurath U. Fleischmann J. (1992). Identification of a gp63 surface glycoprotein in leishmania tarentolae. FEMS Microbiol. Lett 96 (1), 89–92. doi: 10.1111/j.1574-6968.1992.tb05398.x 1526469

[B18] Castelli G. Bruno F. Saieva L. Alessandro R. Galluzzi L. Diotallevi A.. (2019). Exosome secretion by leishmania infantum modulate the chemotactic behavior and cytokinic expression creating an environment permissive for early infection. Exp. Parasitol 198, 39–45. doi: 10.1016/j.exppara.2019.01.014 30716304

[B19] Cavallari C. Ranghino A. Tapparo M. Cedrino M. Figliolini F. Grange C.. (2017). Serum-derived extracellular vesicles (EVs) impact on vascular remodeling and prevent muscle damage in acute hind limb ischemia. Sci. Rep. 7 (1), 1–14. doi: 10.1038/s41598-017-08250-0 28811546 PMC5557987

[B20] Cocucci E. Meldolesi J. (2015). Ectosomes and exosomes: shedding the confusion between extracellular vesicles. Trends Cell Biol. 25 (6), 364–372. doi: 10.1016/j.tcb.2015.01.004 25683921

[B21] Contreras I. Gómez M. A. Nguyen O. Shio M. T. McMaster R. W. Olivier M. (2010). Leishmania-induced inactivation of the macrophage transcription factor AP-1 is mediated by the parasite metalloprotease GP63. PloS Pathogens 6 (10), e1001148. doi: 10.1371/journal.ppat.1001148 20976196 PMC2954837

[B22] Corrales R. M. Sereno D. Mathieu-Daudé F. (2010). Deciphering the leishmania exoproteome: what we know and what we can learn. FEMS Immunol. Med. Microbiol. 58 (1), 27–38. doi: 10.1111/j.1574-695X.2009.00608.x 19807787

[B23] Cuervo P. De Jesus J. B. Saboia-Vahia L. Mendonça-Lima L. Domont G. B. Cupolillo E. (2009). Proteomic characterization of the released/secreted proteins of leishmania (Viannia) braziliensis promastigotes. J. Proteomics 73 (1), 79–92. doi: 10.1016/j.jprot.2009.08.006 19703603

[B24] Dayakar A. Chandrasekaran S. Kuchipudi S. V. Kalangi S. K. (2019). Cytokines: key determinants of resistance or disease progression in visceral leishmaniasis: opportunities for novel diagnostics and immunotherapy. Front. Immunol. 670. doi: 10.3389/fimmu.2019.00670 PMC6459942 31024534

[B25] Dong G. Olivier M. (2019). Modulation of host-pathogen communication by extracellular vesicles (EVs) of the protozoan parasite leishmania. Front. Cell. Infect Microbiol. 9, 100. doi: 10.3389/fcimb.2019.00100 31032233 PMC6470181

[B26] Dong G. Wagner V. Minguez-Menendez A. Fernandez-Prada C. Olivier M. (2021). Extracellular vesicles and leishmaniasis: Current knowledge and promising avenues for future development. Mol. Immunol. 135, 73–83. doi: 10.1016/j.molimm.2021.04.003 33873096

[B27] Douanne N. Dong G. Douanne M. Olivier M. Fernandez-Prada C. (2020). Unravelling the proteomic signature of extracellular vesicles released by drug-resistant leishmania infantum parasites. PloS Negl. Trop. Diseases 14 (7), e0008439. doi: 10.1371/journal.pntd.0008439 PMC7365475 32628683

[B28] Eitan E. Zhang S. Witwer K. W. Mattson M. P. (2015). Extracellular vesicle–depleted fetal bovine and human sera have reduced capacity to support cell growth. J. Extracell Vesicles 4 (1), 26373. doi: 10.3402/jev.v4.26373 25819213 PMC4376846

[B29] Gannavaram S. Bhattacharya P. Siddiqui A. Ismail N. Madhavan S. Nakhasi H. L. (2019). miR-21 expression determines the early vaccine immunity induced by LdCen–/– immunization. Front. Immunol. 10, 2273. doi: 10.3389/fimmu.2019.02273 31608064 PMC6769120

[B30] Geiger A. Hirtz C. Bécue T. Bellard E. Centeno D. Gargani D.. (2010). Exocytosis and protein secretion in trypanosoma. BMC Microbiol. 10 (1), 1–17. doi: 10.1186/1471-2180-10-20 20102621 PMC3224696

[B31] Gessani S. Belardelli F. (1998). IFN-γ expression in macrophages and its possible biological significance. Cytokine Growth Factor Rev. 9 (2), 117–123. doi: 10.1016/S1359-6101(98)00007-0 9754706

[B32] Gimona M. Pachler K. Laner-Plamberger S. Schallmoser K. Rohde E. (2017). Manufacturing of human extracellular vesicle-based therapeutics for clinical use. Int. J. Mol. Sci. 18 (6), 1190. doi: 10.3390/ijms18061190 PMC5486013 28587212

[B33] Gioseffi A. Hamerly T. Van K. Zhang N. Dinglasan R. R. Yates P. A.. (2020). Leishmania-infected macrophages release extracellular vesicles that can promote lesion development. Life Sci. Alliance 3 (12), e202000742. doi: 10.26508/lsa.202000742 33122174 PMC7652379

[B34] Gudbergsson J. M. Johnsen K. B. Skov M. N. Duroux M. (2016). Systematic review of factors influencing extracellular vesicle yield from cell cultures. Cytotechnology 68 (4), 579–592. doi: 10.1007/s10616-015-9913-6 26433593 PMC4960200

[B35] Hassani K. Antoniak E. Jardim A. Olivier M. (2011). Temperature-induced protein secretion by leishmania mexicana modulates macrophage signalling and function. PloS One 6 (5), e18724. doi: 10.1371/journal.pone.0018724 21559274 PMC3086886

[B36] Heirwegh E. MacLean E. He J. Kamhawi S. Sagan S. M. Olivier M. (2021). Sandfly fever Sicilian virus-leishmania major co-infection modulates innate inflammatory response favoring myeloid cell infections and skin hyperinflammation. PloS Negl. Trop. Diseases 15 (7), e0009638. doi: 10.1371/journal.pntd.0009638 PMC8341699 34310619

[B37] Isnard A. Shio M. T. Olivier M. (2012). Impact of leishmania metalloprotease GP63 on macrophage signaling. Front. Cell. Infect Microbiol. 2, 72. doi: 10.3389/fcimb.2012.00072 22919663 PMC3417651

[B38] Kaneko N. Kurata M. Yamamoto T. Morikawa S. Masumoto J. (2019). The role of interleukin-1 in general pathology. Inflammation Regeneration 39 (1), 1–16. doi: 10.1186/s41232-019-0101-5 31182982 PMC6551897

[B39] Katebi A. Gholami E. Taheri T. Zahedifard F. Habibzadeh S. Taslimi Y.. (2015). Leishmania tarentolae secreting the sand fly salivary antigen PpSP15 confers protection against leishmania major infection in a susceptible BALB/c mice model. Mol. Immunol. 67 (2), 501–511. doi: 10.1016/j.molimm.2015.08.001 26298575

[B40] Khadir F. Shaler C. R. Oryan A. Rudak P. T. Mazzuca D. M. Taheri T.. (2018). Therapeutic control of leishmaniasis by inhibitors of the mammalian target of rapamycin. PloS Negl. Trop. Diseases 12 (8), e0006701. doi: 10.1371/journal.pntd.0006701 PMC6122837 30133440

[B41] Kima P. E. Soong L. (2013). Interferon gamma in leishmaniasis. Front. Immunol. 4, 156. doi: 10.3389/fimmu.2013.00156 23801993 PMC3685816

[B42] Kraaij M. D. Vereyken E. J. Leenen P. J. van den Bosch T. P. Rezaee F. Betjes M. G.. (2014). Human monocytes produce interferon-gamma upon stimulation with LPS. Cytokine 67 (1), 7–12. doi: 10.1016/j.cyto.2014.02.001 24680476

[B43] Lambertz U. Ovando M. E. O. Vasconcelos E. J. Unrau P. J. Myler P. J. Reiner N. E. (2015). Small RNAs derived from tRNAs and rRNAs are highly enriched in exosomes from both old and new world leishmania providing evidence for conserved exosomal RNA packaging. BMC Genomics 16 (1), 1–26. doi: 10.1186/s12864-015-1260-7 25764986 PMC4352550

[B44] Li J. Lee Y. Johansson H. J. Mäger I. Vader P. Nordin J. Z.. (2015). Serum-free culture alters the quantity and protein composition of neuroblastoma-derived extracellular vesicles. J. Extracell Vesicles 4 (1), 26883. doi: 10.3402/jev.v4.26883 26022510 PMC4447833

[B45] Lobb R. J. Becker M. Wen Wen S. Wong C. S. Wiegmans A. P. Leimgruber A.. (2015). Optimized exosome isolation protocol for cell culture supernatant and human plasma. J. Extracell Vesicles 4 (1), 27031. doi: 10.3402/jev.v4.27031 26194179 PMC4507751

[B46] Maas S. L. Breakefield X. O. Weaver A. M. (2017). Extracellular vesicles: unique intercellular delivery vehicles. Trends Cell Biol. 27 (3), 172–188. doi: 10.1016/j.tcb.2016.11.003 27979573 PMC5318253

[B47] Mendoza-Roldan J. A. Latrofa M. S. Iatta R. RS Manoj R. Panarese R. Annoscia G.. (2021). Detection of leishmania tarentolae in lizards, sand flies and dogs in southern Italy, where leishmania infantum is endemic: hindrances and opportunities. Parasites Vectors 14 (1), 1–12. doi: 10.1186/s13071-021-04973-2 34493323 PMC8423600

[B48] Meng F. Lowell C. A. (1997). Lipopolysaccharide (LPS)-induced macrophage activation and signal transduction in the absence of src-family kinases hck, fgr, and Lyn. J. Exp. Med. 185 (9), 1661–1670. doi: 10.1084/jem.185.9.1661 9151903 PMC2196288

[B49] Mizbani A. Taheri T. Zahedifard F. Taslimi Y. Azizi H. Azadmanesh K.. (2009). Recombinant leishmania tarentolae expressing the A2 virulence gene as a novel candidate vaccine against visceral leishmaniasis. Vaccine 28 (1), 53–62. doi: 10.1016/j.vaccine.2009.09.114 19818721

[B50] Niimi T. (2016). Leishmania tarentolae for the production of multi-subunit complexes. Advanced Technol. Protein Complex Production Characterization 896, 155–165. doi: 10.1007/978-3-319-27216-0_10 27165324

[B51] Nogueira P. M. de Menezes-Neto A. Borges V. M. Descoteaux A. Torrecilhas A. C. Xander P.. (2020). Immunomodulatory properties of leishmania extracellular vesicles during host-parasite interaction: differential activation of TLRs and NF-κB translocation by dermotropic and viscerotropic species. Front. Cell. Infect Microbiol. 10, 380. doi: 10.3389/fcimb.2020.00380 32850481 PMC7403210

[B52] Raymond F. Boisvert S. Roy G. Ritt J.-F. Legare D. Isnard A.. (2012). Genome sequencing of the lizard parasite leishmania tarentolae reveals loss of genes associated to the intracellular stage of human pathogenic species. Nucleic Acids Res. 40 (3), 1131–1147. doi: 10.1093/nar/gkr834 21998295 PMC3273817

[B53] Saljoughian N. Taheri T. Zahedifard F. Taslimi Y. Doustdari F. Bolhassani A.. (2013). Development of novel prime-boost strategies based on a tri-gene fusion recombinant l. tarentolae vaccine against experimental murine visceral leishmaniasis. PloS Negl. Trop. Dis. 7 (4), e2174. doi: 10.1371/journal.pntd.0002174 23638195 PMC3630202

[B54] Seif S. Kazemi F. Gholami E. Seyed N. Taslimi Y. Habibzadeh S.. (2016). EGFP reporter protein: its immunogenicity in leishmania-infected BALB/c mice. Appl. Microbiol. Biotechnol. 100 (9), 3923–3934. doi: 10.1007/s00253-015-7201-1 26685673

[B55] Silverman J. M. Chan S. K. Robinson D. P. Dwyer D. M. Nandan D. Foster L. J.. (2008). Proteomic analysis of the secretome of leishmania donovani. Genome Biol. 9 (2), 1–21. doi: 10.1186/gb-2008-9-2-r35 PMC2374696 18282296

[B56] Silverman J. M. Clos J. de'Oliveira C. C. Shirvani O. Fang Y. Wang C.. (2010). An exosome-based secretion pathway is responsible for protein export from leishmania and communication with macrophages. J. Cell Sci 123 (6), 842–852. doi: 10.1242/jcs.056465 20159964

[B57] Silverman J. M. Clos J. Horakova E. Wang A. Y. Wiesgigl M. Kelly I.. (2010). Leishmania exosomes modulate innate and adaptive immune responses through effects on monocytes and dendritic cells. J. Immunol. 185 (9), 5011–5022. doi: 10.4049/jimmunol.1000541 20881185

[B58] Silverman J. M. Reiner N. E. (2012). Leishmania exosomes deliver preemptive strikes to create an environment permissive for early infection. Front. Cell. Infect Microbiol. 1, 26. doi: 10.3389/fcimb.2011.00026 22919591 PMC3417360

[B59] Sun L. Wang H.-X. Zhu X.-J. Wu P.-H. Chen W.-Q. Zou P.. (2014). Serum deprivation elevates the levels of microvesicles with different size distributions and selectively enriched proteins in human myeloma cells *in vitro* . Acta Pharmacol Sinica 35 (3), 381–393. doi: 10.1038/aps.2013.166 PMC4647891 24374813

[B60] Szempruch A. J. Sykes S. E. Kieft R. Dennison L. Becker A. C. Gartrell A.. (2016). Extracellular vesicles from trypanosoma brucei mediate virulence factor transfer and cause host anemia. Cell 164 (1-2), 246–257. doi: 10.1016/j.cell.2015.11.051 26771494 PMC4715261

[B61] Théry C. Ostrowski M. Segura E. (2009). Membrane vesicles as conveyors of immune responses. Nat. Rev. Immunol. 9 (8), 581–593. doi: 10.1038/nri2567 19498381

[B62] Théry C. Witwer K. W. Aikawa E. Alcaraz M. J. Anderson J. D. Andriantsitohaina R.. (2018). Minimal information for studies of extracellular vesicles 2018 (MISEV2018): a position statement of the international society for extracellular vesicles and update of the MISEV2014 guidelines. J. Extracell Vesicles 7 (1), 1535750. doi: 10.1080/20013078.2018.1535750 30637094 PMC6322352

[B63] Torrecilhas A. C. Soares R. P. Schenkman S. Fernández-Prada C. Olivier M. (2020). Extracellular vesicles in trypanosomatids: host cell communication. Front. Cell. Infect Microbiol. 10. doi: 10.3389/fcimb.2020.602502 PMC7767885 33381465

[B64] Torrecilhas A. C. T. Tonelli R. R. Pavanelli W. R. da Silva J. S. Schumacher R. I. de Souza W.. (2009). Trypanosoma cruzi: parasite shed vesicles increase heart parasitism and generate an intense inflammatory response. Microbes Infect 11 (1), 29–39. doi: 10.1016/j.micinf.2008.10.003 19028594

[B65] Valdivia H. O. Scholte L. L. Oliveira G. Gabaldón T. Bartholomeu D. C. (2015). The leishmania metaphylome: a comprehensive survey of leishmania protein phylogenetic relationships. BMC Genomics 16 (1), 1–13. doi: 10.1186/s12864-015-2091-2 26518129 PMC4628237

[B66] Webber J. Clayton A. (2013). How pure are your vesicles? J. Extracell Vesicles 2 (1), 19861. doi: 10.3402/jev.v2i0.19861 PMC3760653 24009896

[B67] Zahedifard F. Gholami E. Taheri T. Taslimi Y. Doustdari F. (2014). Enhanced protective efficacy of nonpathogenic recombinant leishmania. PLoS Negl Trop Dis 8 (3), e2751. doi: 10.1371/journal.pntd.0002751 24675711 PMC3967951

