# Supplementary material for: Isolation, characterization, and functional study of extracellular vesicles derived from Leishmania tarentolae
Source: Front Cell Infect Microbiol. 2022 Aug 3;12:921410. doi: 10.3389/fcimb.2022.921410 (PMC9381964; doi:10.3389/fcimb.2022.921410)
Supplement: Supplementary file 1 [file DataSheet_1.docx]

**Supplementary Figures and Tables**


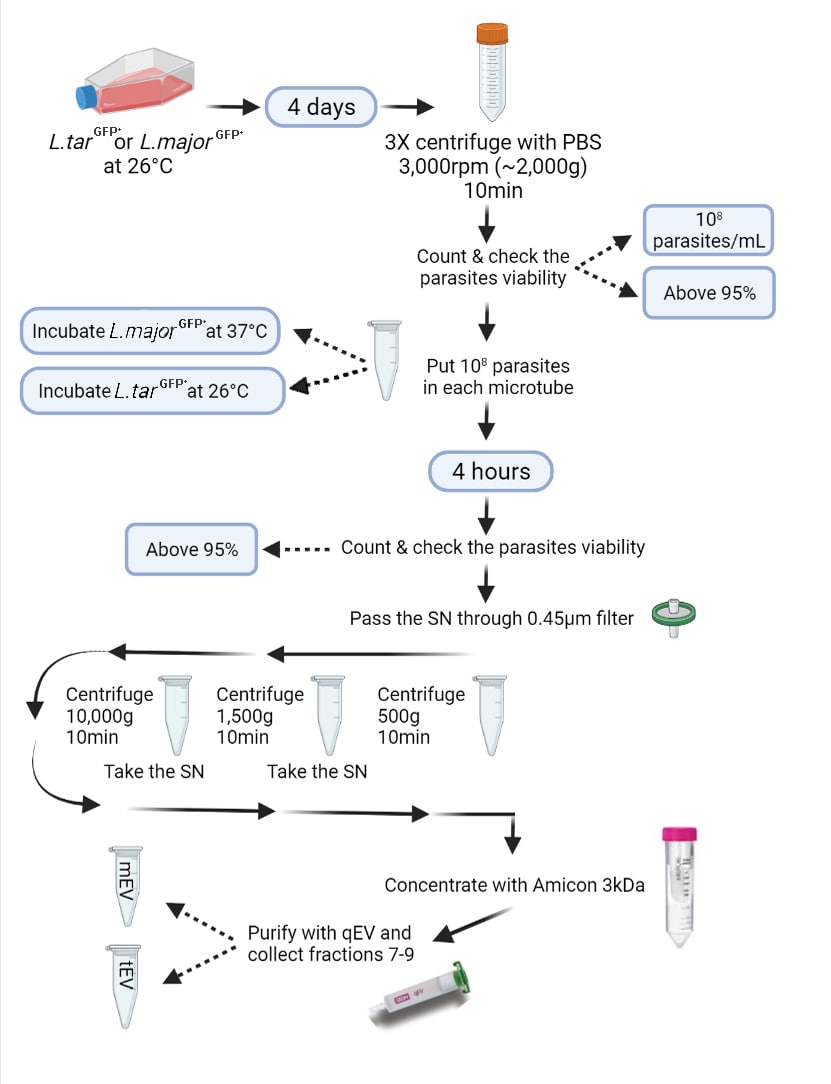


**Supplementary Figure 1.** Workflow for isolation of EVs in the present study. The culture media of the late-log phase parasites were replaced with EV-depleted FBS (EFBS) and after the counting and viability assessment using Resazurin, parasites were incubated at given temperatures. After four hours, the number and viability of parasites were measured again in order to make sure about viability of the parasites. Then, the supernatant was passed through a 0.45µm syringe filter prior to the three-step serial centrifugation. The final supernatant was concentrated and purified using Amicon 3kDa centrifugal filter and qEV SEC column, respectively**.**

**
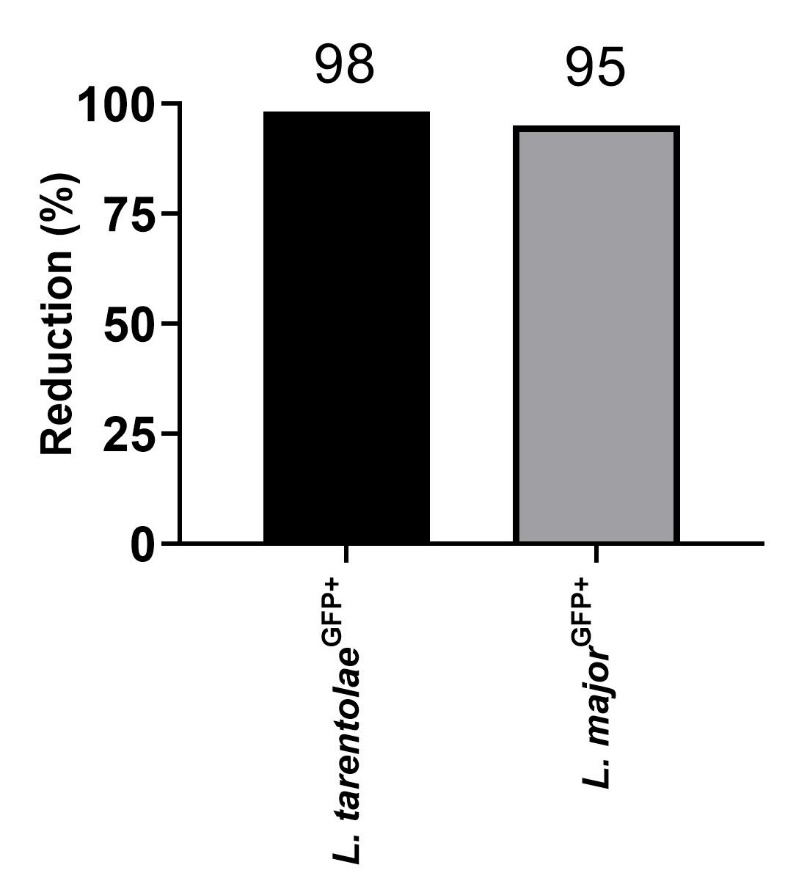
**

**Supplementary Figure 2**. Reduction rate of Resazurin in both species after 4 hours of incubation in serum-free media. In order to rule out the possibility of forming apoptotic bodies by the parasites, a viability assessment was done on parasites from which the studied EVs were isolated. The bar chart above shows that both species have reduced at least 95% of Resazurin solution. The reduction rate is directly correlated with their viability.

**Supplementary Table 1: Calculation of the reduction rate and viability with Resazurin:**

In order to calculate the percentage of reduction rate or viability, we used the below formula using these parameters according to the Manufacturer’s instructions:

- Molar extinction coefficients based on the setup wavelength on the ELISA reader:

|  | Ɛ_RED_ | Ɛ_OX_ |
| --- | --- | --- |
| 540nm | 104395 | 47619 |
| 630nm | 5494 | 34798 |

- In accordance with the wavelength and molar extinctions mentioned above, we calculated the reduction rate and viability using these equations:

%Reduction=$\frac{\left( \varepsilon_{OX} \right)\lambda_{2}A\lambda_{1}-\left( \varepsilon_{OX} \right)\lambda_{1}A\lambda_{2}}{\left( \varepsilon_{RED} \right)\lambda_{1}A^{'}\lambda_{2}-(\varepsilon_{RED})\lambda_{2}A^{'}\lambda_{1}}\times100$

%Viability=$\frac{\left( \varepsilon_{OX} \right)\lambda_{2}A\lambda_{1}-\left( \varepsilon_{OX} \right)\lambda_{1}A\lambda_{2}}{\left( \varepsilon_{OX} \right)\lambda_{2}A^{^{\circ}}\lambda_{1}-(\varepsilon_{OX})\lambda_{1}A^{^{\circ}}2}\times100$

Where

| λ_1_=540nm | λ_2_=630nm |
| --- | --- |
| (Ɛ_OX_)λ_1_=47619 | (Ɛ_OX_)λ_2_=34798 |
| (Ɛ_RED_)λ_1_=104395 | (Ɛ_RED_)λ_2_=5494 |
| A=absorbance of test wells | A’=absorbance of negative control well. |
| A°=absorbance of positive growth control well |  |

**
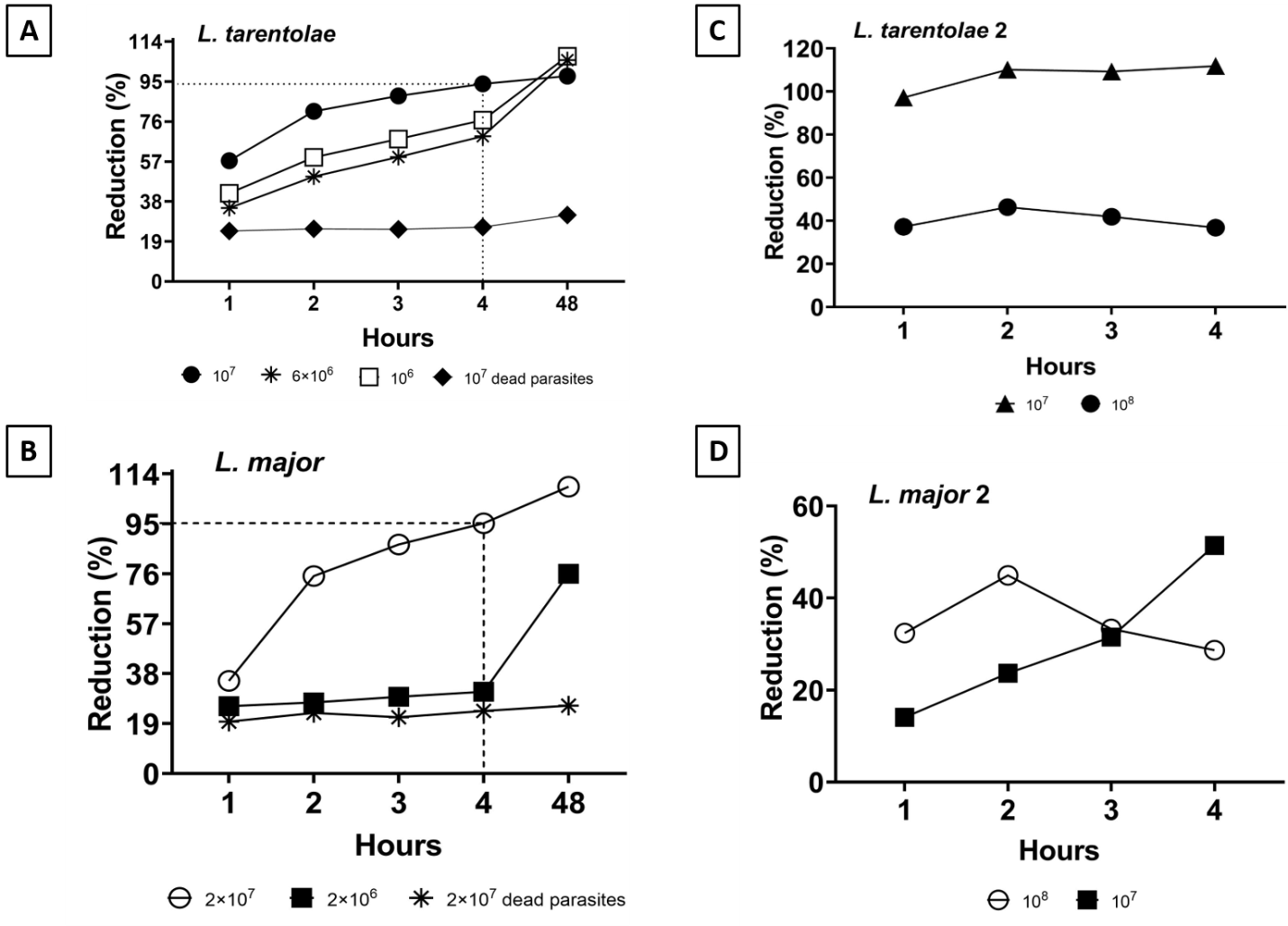
**

**Supplementary Figure 3.** Total number of logarithmic parasites required for viability assessment using Resazurin solution. **A)** *L. tarentolae*^GFP+^ and *L. major*^GFP+^ with different concentrations were subjected to the Resazurin solution at given periods in presence of dead parasites as negative control. The total number of 10^7^ parasites was the best indicator of their viability. **B)** *L. tarentolae*^GFP+^ and *L. major*^GFP+^ with two different concentrations. 10^7^ parasites per well was selected according to the results of panel A, and 10^8^ parasites per well was selected to check whether a higher parasite number in each well would result in higher viability percentage or not. Again, the best results were obtained from wells which 10^7^ parasites were cultured. According to two individual experiments on *L. tarentolae*^GFP+^ and *L. major*^GFP+^ the best concentration of parasites is 10^7^ parasites (in total) for viability tests.

**Supplementary Figure 4.** In order to confirm the isolated EVs from *Leishmania tarentolae* were produced by the said parasites, another batch of both species were simultaneously incubated for 4 hours in a serum-free culture media in presence of Thiomresol. As a results, the parasites were killed and fixed at the beginning of the incubation and no vesicular structures were observed at the end of the incubation using FE-SEM. **A)** *L. major*^GFP+^ **B)** *L. tarentolae*^GFP+^.


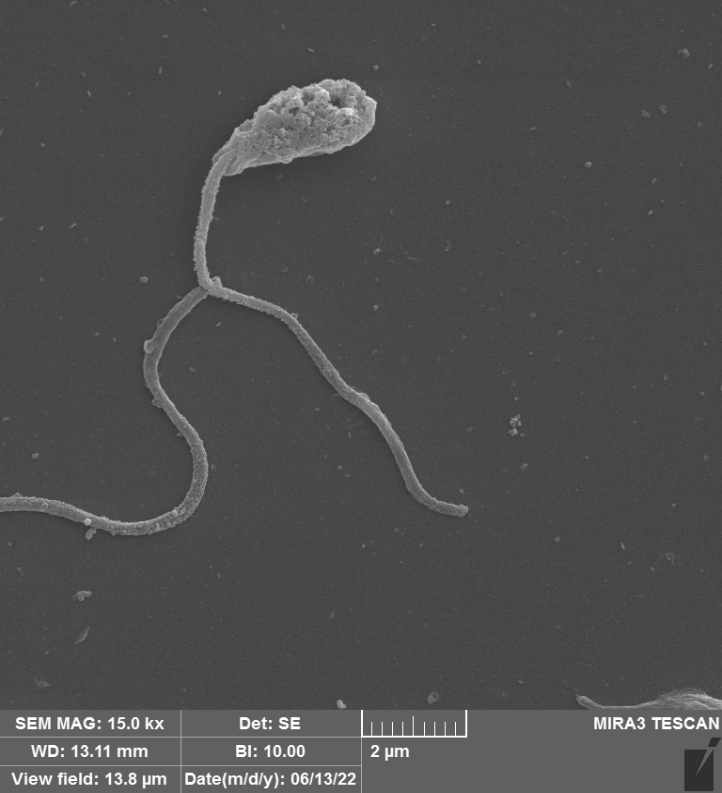


**A**

**B**

**A**

**B**


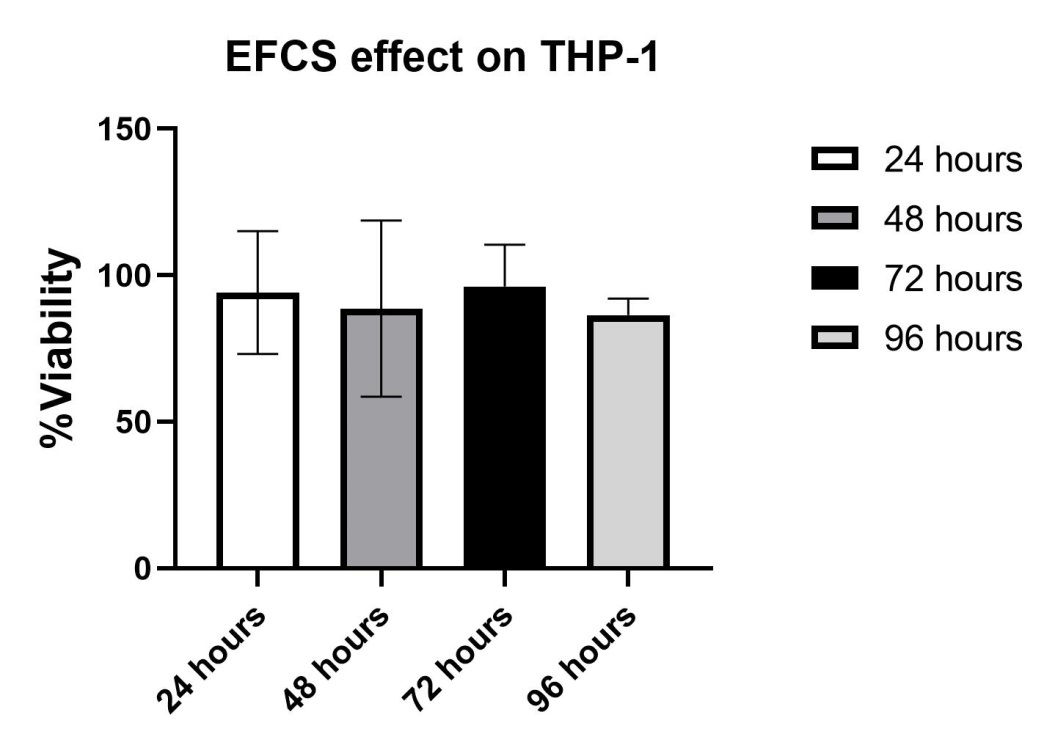

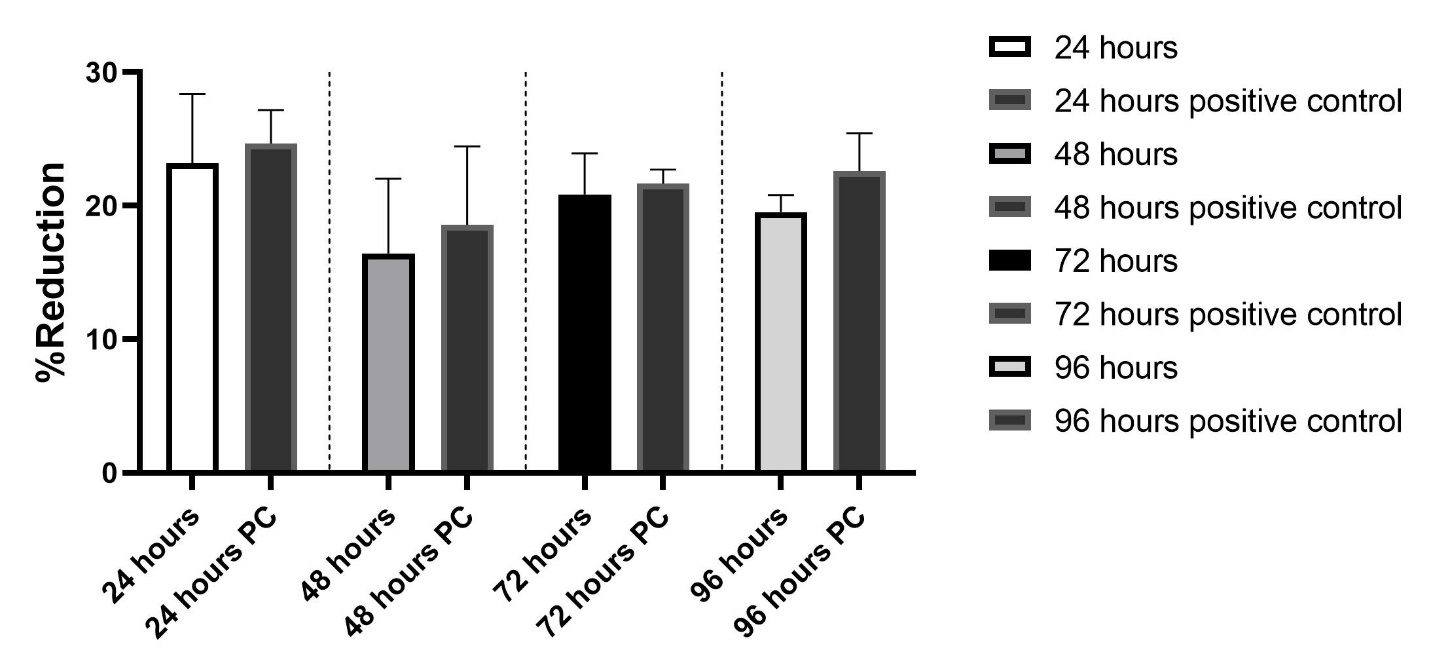


**Supplementary Figure 5**. Since for most of the experiments in this study the fetal bovine serum of the culture medium had to be free of EVs, it was important to know the effect of such culture medium on THP-1 macrophages. Therefore, a viability test was performed using Resazurin to evaluate the effect of this culture medium at different time intervals. **(A)** The effect of EV-depleted FBS on the viability of THP-1 cells at different time intervals. **(B)** The effect of EV-depleted FBS and regular FBS (as positive control) on the Resazurin reduction rate of THP-1 cells at different time intervals. THP-1 macrophages were cultured in a 96-well plate and Resazurin solution was added to the well every day and after four hours of incubation their viability and reduction rate were assessed. After calculating the amount of light absorption in the wells for the period of interest, statistical analyzes did not show significant differences among the groups.
